# Single‐Cell Atlas of Subchondral Bone Marrow Lesions Reveals Proteostasis Dysfunction as a Druggable Mechanism for Early Osteoarthritis

**DOI:** 10.1002/advs.202516720

**Published:** 2026-02-13

**Authors:** Hailun Xu, Ting He, Yu Qian, Jia Li, Bowei Ni, Xiaojie Xu, Huanbo Wang, Peng Wang, Guoqing Cao, Siqi Ying, Guangyu Ding, Rong Wang, Zenghui Gu, Wei Li, Zhentao Man, Houfeng Zheng, Zhaohua Zhu, Zhen Li, Qingjun Meng, Chao Zheng, Liu Yang

**Affiliations:** ^1^ Institute of Orthopedic Surgery Xijing Hospital Fourth Military Medical University Xi'an P. R. China; ^2^ Shandong Provincial Hospital Affiliated to Shandong First Medical University Jinan P. R. China; ^3^ Suzhou Laboratory of Precision Health and Data Science the Second Affiliated Hospital of Soochow University Suzhou Jiangsu P. R. China; ^4^ Institute of Health Data Science Soochow University Suzhou Jiangsu P. R. China; ^5^ Division of Orthopaedic Surgery Department of Orthopedics Nanfang Hospital Southern Medical University Guangzhou Guangdong P. R. China; ^6^ Department of Orthopedics Air Force Hospital of Eastern Theater Command Nanjing Jiangsu P. R. China; ^7^ The 989th Hospital of the Joint Logistics Support Force of the Chinese People's Liberation Army Luoyang Henan P. R. China; ^8^ Clinical Research Centre Zhujiang Hospital Southern Medical University Guangzhou Guangdong P. R. China; ^9^ Department of Rheumatology Royal North Shore Hospital and Sydney Musculoskeletal Health Kolling Institute University of Sydney Sydney Australia; ^10^ AO Research Institute Davos Davos Platz Switzerland; ^11^ Faculty of Biology Medicine and Health University of Manchester Manchester UK; ^12^ Medical Research Institute Northwestern Polytechnical University Xi'an P. R. China

**Keywords:** bone marrow lesions, chondrocyte hypertrophy, misfolded collagen, osteoarthritis, single‐cell RNA sequencing

## Abstract

Subchondral bone marrow lesions (BMLs) constitute a pathognomonic imaging feature of both incipient and progressive osteoarthritis (OA). However, the pathological features and molecular mechanisms underlying BMLs remain poorly characterized. Here, we innovatively established and dynamically characterized a standardized mouse model, simulating human BMLs on magnetic resonance imaging (MRI) that correlates significantly with cartilage degeneration, while revealing substantial aberrant bone matrix accumulation within BML regions. By establishing an osteochondral single‐cell atlas of mouse knee joints during BML development, we found that proteasome dysfunction and abnormal secretion of misfolded collagen, driven by dysregulated heat shock protein 70 (HSP70) and deubiquitinase 19 (USP19) expression in osteoarthritic subchondral osteoblasts, constitute a key mechanism of BML formation. Furthermore, cell‐cell communication and *Col10a1‐Cre; R26^tdt+^* fate‐mapping analyses uncovered that osteoblast‐secreted WNT5A mediates crosstalk with hypertrophic chondrocytes (HTCs), accelerating their hypertrophy and cell death. Critically, pharmacological HSP70 targeting by TRC051384 inhibited collagen misfolding/secretion, preventing BML formation. Notably, results of Mendelian randomization demonstrated a significant correlation between proteasome gene expression and OA risk in humans, further supporting a potential role of proteasome dysfunction in BML pathogenesis. Collectively, these data reveal mechanisms underlying BML formation and therapeutic targets for early OA intervention.

## Graphical abstract

This study reveals that abnormal mechanical stress downregulates the expression of HSP70 and impairs proteasome function in osteoarthritic bone cells, leading to misfolded collagen I accumulation and ER stress. When intracellular proteostasis capacity is exceeded, USP19 mediates the secretion of misfolded proteins into the extracellular space, promoting aberrant bone matrix deposition and driving BML formation.

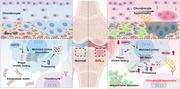

## 1 Introduction

Osteoarthritis (OA) is a common chronic degenerative disease, affecting approximately 590 million people globally and serving as a leading cause of disability [1, 2]. Currently, no strategies effectively prevent or halt OA progression, particularly during the early reversible stage of damage [3]. Emerging evidence suggests that subchondral bone lesions may precede and trigger cartilage damage in OA [4, 5], with bone marrow lesions (BMLs) representing an emerging imaging biomarker in subchondral bone [6, 7]. However, research on early‐stage BML has not been conducted thus far due to the inability to obtain human samples and the lack of effective, stable animal models.

BMLs are detectable on magnetic resonance imaging (MRI) using fluid‐sensitive, fat‐suppressed sequences in the T2 phase [8]. As a metabolically active region within subchondral bone, BMLs are recognized as microdamage of bone matrix induced by aberrant mechanical stress, exhibiting dynamic alterations, with over half of lesions showing progressive deterioration [9]. In the histopathology level, bone trabecular thickening, vascular proliferation, fibrosis, and adipocyte infiltration have been observed in post‐surgical BMLs [10]. Furthermore, in the clinical research level, the BML is strongly associated with joint pain, cartilage defect, and the risk of joint replacement [11]. Importantly, BMLs have emerged as an early imaging biomarker in OA, as they often precede radiographic cartilage loss and can predict structural progression and symptom severity [12]. However, the early pathological phenotypes and pathogenesis of BMLs and their role in driving structural deterioration in OA have not been reported.

Previous studies have performed scRNA‐seq analyses on bone and cartilage in OA. For instance, a single‐cell atlas of subchondral bone tissue from late‐stage OA patients elucidated the roles of distinct endothelial cell and osteoblast populations in OA progression [13]. Additionally, single‐cell atlases derived from human and mouse OA cartilage, respectively, revealed heterogeneous characteristics among chondrocyte populations [14, 15]. Notably, Sun et al. identified a significant correlation between hypertrophic chondrocytes (HTCs) and OA in a human cartilage single‐cell atlas but have yet to elucidate the specific triggers for chondrocyte hypertrophy [16]. Additionally, current single‐cell studies on OA have predominantly focused on isolated cartilage or bone tissues, failing to dissect the bone‐cartilage interaction mechanisms within the intact osteochondral unit, particularly during BML development. Thus, constructing a composite osteochondral single‐cell atlas of the BML period is essential for elucidating the mechanisms of BML development and bone‐cartilage interactions.

Here, we established a mouse model of simulating early‐stage BML using anterior cruciate ligament transection (ACLT) combined with running wheel exercise (Running), uncovering the pathologic phenotype of aberrant bone accumulation in BMLs. Concurrently, by constructing a composite osteochondral single‐cell atlas at the BML development, we demonstrated that downregulation of HSP70 in osteoarthritic bone cells causes a pathology characterized by misfolded COLI, while USP19 facilitates the secretion of misfolded proteins to the extracellular space, leading to the accumulation of aberrant bone matrix. Through cell‐cell communication analyses, we identified WNT5A secreted by osteoarthritic bone cells as the primary driver of chondrocyte hypertrophy. Last, we demonstrated a remarkable correlation between proteasome dysfunction and OA in humans, further confirming the crucial role of the proteasome in the BMLs development. These findings provide multidimensional therapeutic targets for early‐stage and progressive BML intervention.

## 2 Results

### 2.1 Modeling the Onset and Progression of Human BMLs in Mice Undergoing Post‐Operative Wheel Running

**FIGURE 1:**
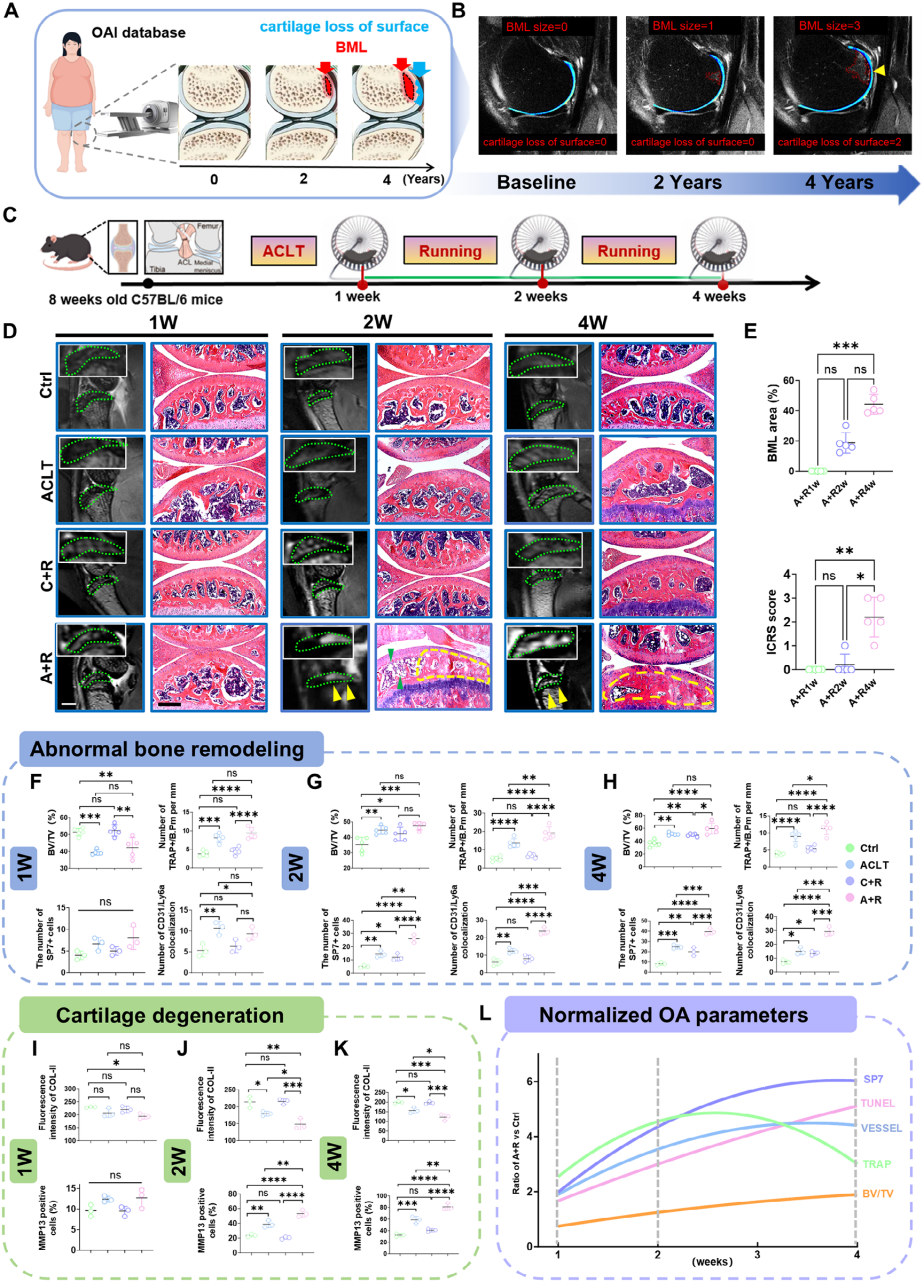
Recapitulating human BML development in mice performing post‐operative wheel exercise. (A and B) Schematic diagram (A) and representative images (B) of the BML development of an individual in the OAI database at baseline 2 and 4 years. (C) Schematic diagram of constructing the combined anterior cruciate ligament transection (ACLT) and forced wheel running model (A+R). (D) Representative images of 9.4T magnetic resonance imaging (MRI) sequence of T2 phase fat suppression and HE staining (green dotted lines: subchondral bone region; yellow dashed line and arrows: BMLs area; green arrows: adipocytes). scale bars = 200 µm (HE); scale bars = 1 mm (MRI). (E) Quantification of BML area in subchondral bone and International Cartilage Repair Society (ICRS) score of cartilage (n = 5). (F–H) Quantification of bone volume/trabecular bone volume ratio (BV/TV) and staining results of tartrate‐resistant acid phosphatase (TRAP), SP7, and CD31/Ly6a from subchondral bone in the BML model (n = 5 for BV/TV and TRAP; n = 3 for SP7 and CD31/Ly6a). (I‐K) Quantification of immunostaining of COLII and MMP13 from cartilage in the BML model (n = 3). (L) Normalized parameters of the A+R group to controls. Data are shown as the mean ± SD. Statistical significance was assessed using the Kruskal‐Wallis test (E) or one‐way ANOVA (F–K). ^*^ *p* < 0.05, ^**^ *p* < 0.01, ^***^ *p* < 0.001, ^****^ *p* < 0.0001.

As depicted in Figure 1A, we evaluated a 4‐year series of right knee MRI scans from a 57‐year‐old female (data from the Osteoarthritis Initiative study [17]). BML size and cartilage loss of the surface were assessed using the MRI Osteoarthritis Knee Score (MOAKS). We found that BML size progression preceded the progression of cartilage loss of surface (Figure 1B), i.e., BML size led the increase from 0 to 1 (baseline to 2‐year follow‐up), followed by the increase of cartilage loss of surface from 0 to 2 in the next period (2 to 4‐year follow‐ups) (Figure 1B). To validate these findings, we set out to build a post‐operative wheel running model in mice to simulate the onset and progression of human BMLs (Figure 1C). One week post‐modeling, MRI detected no signs of BMLs in any group (Figure 1D). Notably, the A+R group exhibited high‐signal BML lesions on T2 MRI sequences with fat‐suppressed effect at two weeks post‐modeling, with corresponding HE sections showing aberrant bone matrix accumulation within BML regions (Figure 1D). Moreover, aberrant adipocyte accumulation appeared in non‐lesion areas, confirmed by Sudan III staining (Figure S2E). Four weeks post‐modeling, MRI displayed diffuse multifocal BMLs in the A+R group, while histology revealed subchondral abnormal bone matrix accumulation (Figure 1D), demonstrating a spatial consistency with imaging features. Critically, cartilage damage progresses further with the development of BML (Figure 1E), suggesting a correlation between BML and cartilage degeneration.

To dynamically analyze the mouse model of BMLs, we conducted a multifaceted assessment of bone and cartilage at 1, 2, and 4 weeks after modeling (Figures S1A, S2A, S3A). One week post‐modeling, the A+R group exhibited a decrease in trabecular bone density accompanied by an increase in TRAP^+^ osteoclasts compared to controls (Figure 1F; Figure S1B, C), indicating initiation of bone remodeling. Additionally, the expressions of the osteogenic marker SP7 and CD31/Ly6a as the marker for S‐type vessels, a vascular subtype of secondary ossification center, were mildly upregulated (Figure 1F; Figures S1D and S4) [18]. However, the cartilage did not exhibit matrix degradation, chondrocyte apoptosis, and structural damage (Figure 1I; Figure S1E–H), suggesting the early OA stage of this model at 1 week. Two weeks post‐modeling, the A+R group exhibited marked trabecular bone density increase, while TRAP+ osteoclast numbers continued to increase (Figure 1G; Figure S2B, C). Concurrently, SP7 expression and vascular density increased significantly (Figure 1G; Figure S2D), indicating a shift in bone remodeling balance toward bone‐anabolism. In cartilage, COLII expression was decreased, MMP13 and TUNEL expression were increased, yet cartilage did not present obvious structural damages (Figure 1J; Figure S2F–I), suggesting this stage remains within the early reversible stage of OA. Four weeks post‐modeling, the A+R group exhibited plate‐like trabeculae and osteophytes (Figure 1H; Figures S3B and S5). Notably, TRAP+ osteoclasts decreased, while SP7 and vascularity were upregulated (Figure S3C, D), indicating osteogenesis‐dominated remodeling driving sclerosis. Additionally, calcein double‐labeling showed enhanced bone formation in the BML group, evidenced by increased trabecular mineral apposition rate (MAR), mineralized surface/bone surface (MS/BS), and bone formation rate/bone surface (BFR/BS) (Figure S6). Cartilage showed obvious damage, accompanied by increased chondrocyte apoptosis (Figure S3E–H). Collectively, based on multifaceted validation of the A+R model, we constructed a dynamic profile of these changes (Figure 1L; Figure S7), providing a reference standard for investigating the pathogenesis of BMLs.

### 2.2 scRNA‐seq Uncovers Defective Protein Folding and Aberrant Osteochondral Crosstalk in BMLs

**FIGURE 2:**
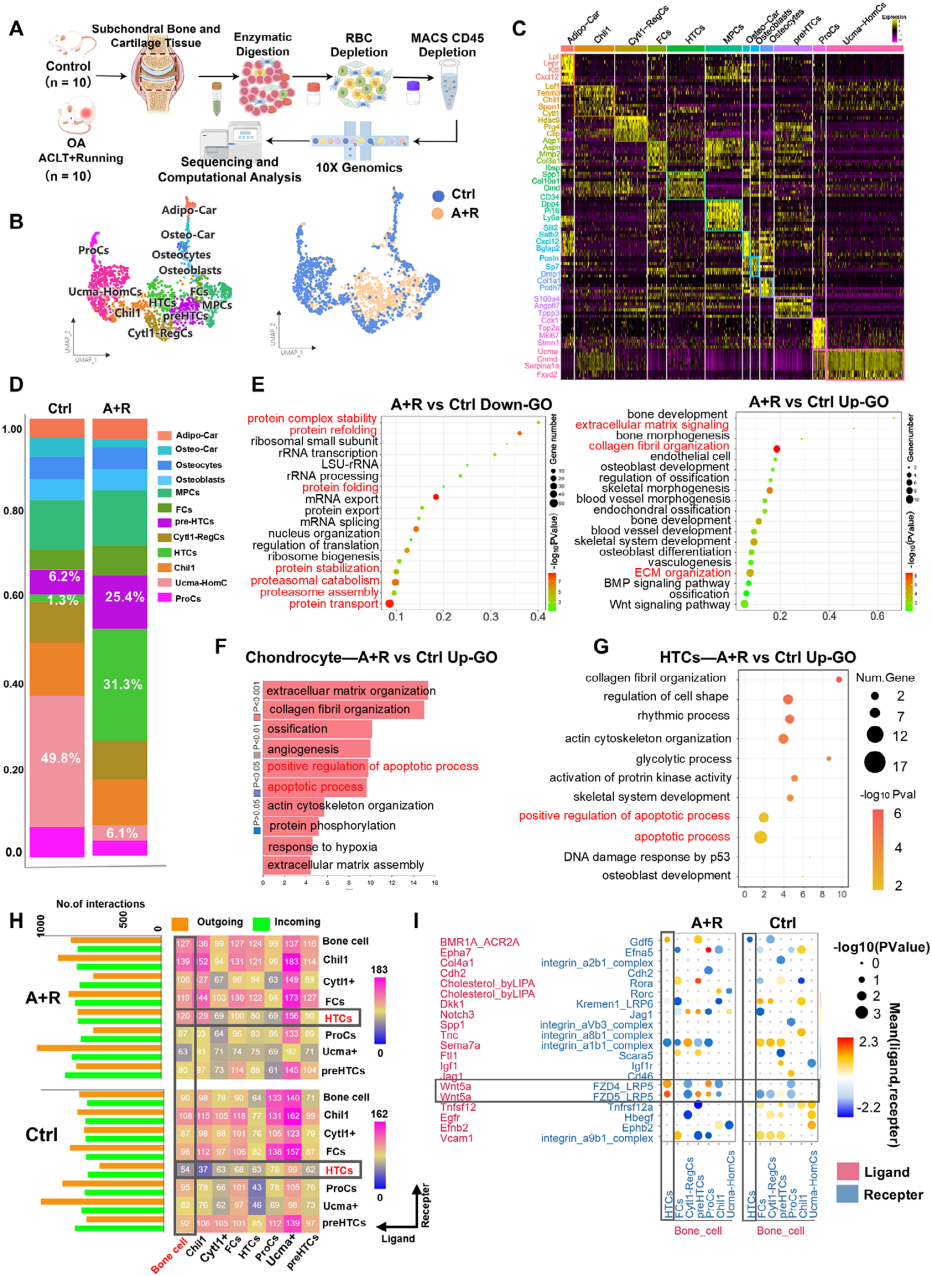
Single‐cell RNA sequencing analysis of osteochondral tissue from BML and control mice was performed at the 2‐week post‐model induction. (A) Schematic of workflows for transcriptomic profiling of mice articular cartilage and subchondral bone using single‐cell RNA sequencing (scRNA‐seq). (B) UMAP plots of cell subpopulations and cell distribution for BML and control groups. (C) Heatmap showing the expression of differentially expressed genes (DEGs) in each cluster defined in (B), with yellow indicating high expression and black indicating low or no expression. (D) Proportion of each cell subpopulation in the BML and control groups. (E) The significant Gene Ontology (GO) terms were enriched by the upregulated and downregulated DEGs in bone cells from BML groups compared to control groups. Bone cells represent osteo‐lineage cells, primarily including osteoblasts and osteocytes. (F) The significant GO terms were enriched by the upregulated DEGs in chondrocytes from the BML groups compared to the control groups. (G) The significant GO terms were enriched by the upregulated DEGs in hypertrophic chondrocytes (HTCs) from BML groups compared to control groups. (H) Interaction strength between cell populations in the BML and control groups. Solid boxes indicate changes in interaction strength between bone cells as ligand sender and the HTCs population as receptor in the BML and control groups. (I) Dot plots illustrate the increased signaling and decreased signaling of ligand‐receptor pairs between the BML and control groups. The color of the dots defines the communication strength of specific signaling pairs between different cell types. The size of the dots defines the *P*‐value of a specific signaling pair between different cell types.

To investigate BMLs induced by post‐operative wheel running in mice at a single‐cell resolution, we subjected tibial plateaus and femoral condyles from hind limbs to scRNA‐seq analyses (Figure 2A). A total of 14,291 cells were obtained from the BML and control groups, with 13,446 cells retained after stringent filtration (Figure S8A). The osteochondral cell lineage was then isolated for subsequent analyses, and these cells comprised 12 distinct clusters defined by previously reported markers [14, 15, 16, 19] (Figure 2B, C; Figures S8B and S9). Cartilage‐lineage populations comprised 7 subsets: the proliferative chondrocytes (ProCs), *Ucma*‐positive chondrocytes (*Ucma*‐HomCs), *Chil1*‐positive chondrocytes (*Chil1+*), HTCs, *Cytl1*‐positive regulatory cells (*Cytl1*‐RegCs), pre‐hypertrophic chondrocytes (pre‐HTCs), and fibrochondrocytes (FCs). The results of pseudotime trajectory analyses suggested that *Chil1+*, ProCs, and *Ucma*‐HomCs served as the starting point, transited through intermediate *Cytl1*‐RegCs, and ultimately differentiated into late‐stage pre‐HTCs, HTCs, and FCs (Figure S10C, D). Bone‐lineage populations included osteoblasts expressing *Sp7, Bglap2*, and *Postn*, and mature osteocytes expressing *Dmp1* and *Col1a1*. Two CXC chemokine ligand 12 (CXCL12)–abundant reticular (CAR) cells subtypes functioned as “cytokine reservoirs” [19]. Adipo‐CARs expressed *Lepr, Lpl*, and *Cxcl12*, while Osteo‐CARs shared *Cxcl12* but uniquely expressed neural regulators *Satb2* and *Slit2* (Figure 2C).

To elucidate the pathological mechanisms of BMLs and their impact on overlying cartilage, we compared differences in bone cell populations, chondrocyte populations, and osteochondral crosstalk between the BML and control groups. No significant change in bone‐cell numbers was observed between groups (Figure S10A). However, enrichment analyses of the differentially expressed genes in bone cells between the BML and control groups indicated that genes enriched for collagen assembly and extracellular matrix organization were significantly upregulated, whereas genes for protein folding and stabilization were significantly downregulated by the BML modeling (Figure 2E), suggesting disturbed proteostasis and potential protein misfolding in BMLs. Meanwhile, Kyoto Encyclopedia of Genes and Genomes (KEGG) enrichment results aligned with GO analysis, showing significant upregulation of genes of collagen synthesis and osteogenesis‐related TGF‐β and WNT pathways, while genes enriched for proteasome functional terms were significantly downregulated (Figure S11). In cartilage, proportions of HTCs (1.3% vs. 31.3%), pre‐HTCs (6.2% vs 25.4%), and FCs (4.8% vs 10.3%) increased in the BML group (Figure 2D; Figure S10B), with HTCs showing the most pronounced expansion. The entire chondrocytes derived from the BML group enriched genes related to apoptotic signaling pathways (Figure 2F), while protein translation‐ and RNA splicing‐related genes were downregulated (Figure S12). Notably, the HTCs subpopulation also exhibited a significant enrichment of apoptosis gene expression (Figure 2G), indicating an increase in HTCs death. Results of cell‐cell communication analysis demonstrated that when bone cells served as the sole ligand sender, the interaction strength between bone cells and HTCs increased most markedly in the BML group, implying that bone cells might primarily influence overlying cartilage through HTCs (Figure 2H). Crucially, we identified that bone cells and HTCs communicate via the WNT5A‐FZD4/5‐LRP5 ligand‐receptor pair, suggesting bone‐derived WNT5A as the core signaling molecule mediating osteochondral crosstalk during BML development (Figure 2I). Collectively, these findings indicate that BML formation may be associated with disturbance in proteostasis and protein folding of subchondral bone cells, and bone cell‐derived WNT5A may mediate the increase in chondrocyte hypertrophy and death.

### 2.3 HSP70 Deficiency Drives BML Formation by Causing Extracellular Accumulation of Misfolded Collagen

**FIGURE 3:**
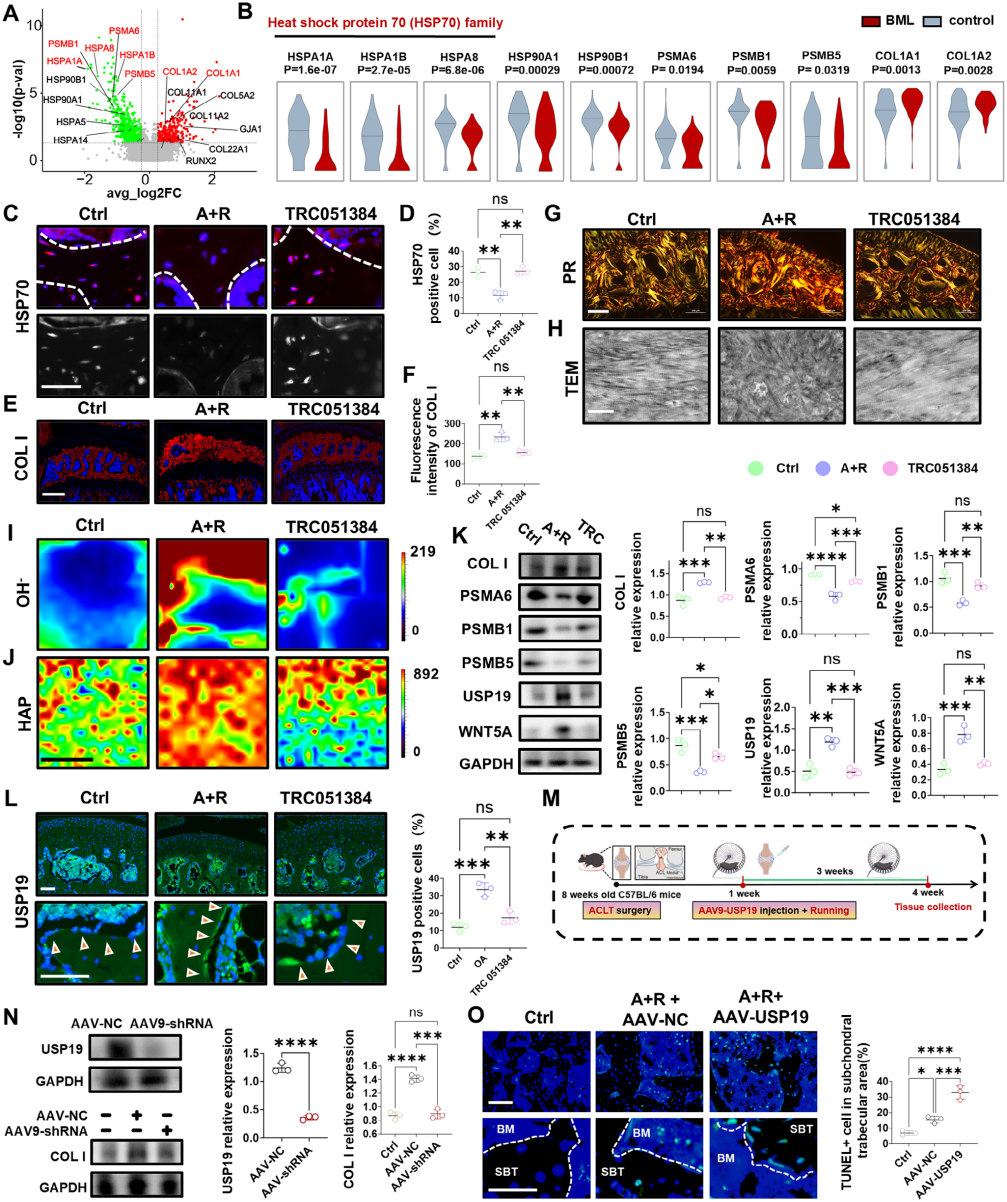
Aberrant extracellular accumulation of misfolded collagen and proteasome dysfunction drives BML formation. (A) Volcano plots of differentially expressed genes in bone cells between the BML versus control groups by pseudobulk analysis of the single‐cell sequencing data. (B) Violin plots of representative gene expression in control and BML groups. (C and D) Representative images (C) and quantification (D) of immunostaining of HSP70 at the 4‐week in control, BML, and treatment groups (n = 3). The white dashed lines mark the junction of the marrow cavity with the trabecular bone. scale bars = 25 um. (E and F) Representative images (E) and quantification (F) of immunostaining of COLI in control, BML, and treatment groups (n = 3). scale bars = 200 um. (G) Representative image of Sirius red staining showing the collagen distribution in control, BML, and treatment groups. scale bars = 100 um. (H) Representative images of transmission electron microscopy showing the morphology and orientation of collagen fibers in control, BML, and treatment groups. scale bars = 500 nm. (I, J) Raman mapping spectra showing the enrichment levels of OH‐ (I) and HAP (J) in the test regions, and the test area in the control groups was a random area of the subchondral bone trabeculae, while the test area in the BML samples was the BML area, with each test area measuring 20 µm × 20 µm. scale bars = 10 um. (K) Western blot analysis and quantification of COLI, PSMB1, PSMB5, PSMA6, USP19, and WNT5A in subchondral bone tissue proteins from control, BML, and treatment groups (n = 3). (L) Representative images and quantification of immunostaining of USP19 in control, BML, and treatment groups (n = 3). Arrows indicate osteoblasts at the margin of bone trabeculae expressing USP19. scale bars = 50 um. (M) Schematic diagram of intra‐articular injections of AAV9‐USP19 to mice receiving BML modeling. (N) Western blot analysis and quantification of the knockdown efficacy of USP19 (top) and the immunofluorescence staining of COLI expression in the extracellular matrix after USP19 knockdown (bottom) (n = 3). (O) Representative images and quantification of staining of TUNEL in control, negative control, and AAV‐USP19 groups (n = 3). The dashed lines indicate the boundary between bone trabeculae and the bone marrow cavity. scale bars = 50 um. Data are shown as the mean ± SD. The difference between the two groups was analyzed by Student's t‐test (N). Multiple group comparison was assessed using one‐way ANOVA (D, F, K, L, N, O). ^*^ *p* < 0.05, ^**^ *p* < 0.01, ^***^ *p* < 0.001, ^****^ *p* < 0.0001.

To further investigate the pathogenesis of BMLs, we analyzed differentially expressed genes in bone cells from the BML and control groups and identified 744 significantly down‐regulated and 367 significantly up‐regulated genes (Figure 3A, B). Downregulated genes were primarily enriched in the heat shock protein family (Hspa1a, Hspa1b, Hspa8, Hsp90), proteasome function (Psma6, Psmb1, Psmb5), with Hspa1a, Hspa1b, and Hspa8 involved in the repair of misfolded proteins [20] while exhibiting anti‐apoptotic and cytoprotective functions [21], suggesting HSP70 deficiency may lead to a protein misfolding‐related pathology; upregulated genes related to collagen formation (Col1a1, Col1a2) and osteogenic/angiogenesis genes (Runx2, Gja1), consistent with the results of GO analysis. To validate the role of HSP70, immunofluorescence staining showed significantly reduced HSP70 expression of the bone cells in the BML group, whereas the HSP70 inducer TRC051384 [22] effectively restored its expression and decreased COLI expression (Figure 3C–F). Meanwhile, we also verified that the expression of HSP90 in the BML group was significantly decreased, which is consistent with the expression of HSP70 (Figure S13). Sirius red staining and transmission electron microscopy (TEM) together demonstrated disordered collagen fiber arrangement and elevated COLI proportion in the BML group, while treated and control groups exhibited regular fiber structures (Figure 3G,H). Moreover, western blot results demonstrated that the activation of the TGFβ‐Smad2/3 pathway may accelerate pathological COLI accumulation (Figure S14). Furthermore, we verified that the WNT5A of BML samples was remarkably upregulated in bone cells and decreased after TRC051384 intervention, implying that decreased HSP70 may promote the secretion of WNT5A by bone cells, thereby affecting HTCs. Considering the bone matrix accumulation in the BML region appeared as hyperintensity on MRI, Raman mapping verified increased levels of hydroxyl (OH‐) and hydroxyapatite (HAP) in the early BML region [23, 24] (Figure 2I,J), suggesting the accumulation of aberrant bone matrix. Interestingly, given the established clinical association between BML and pain, we further assessed calcitonin gene‐related peptide (CGRP) expression in BML areas. Compared with controls, CGRP‐positive nerve fiber density was significantly elevated in the BML group, while TRC051384 intervention markedly reduced CGRP expression (Figure S15).

Accumulation of misfolded proteins could trigger endoplasmic reticulum stress (ERS) and ultimately initiate cell apoptosis [25]. In keeping with this, we found significantly elevated expression of ERS markers ATF6, XBP1, and CHOP in the BML group (Figure S16A, B). Collagen hybridizing peptide (CHP), a probe specifically targeting misfolded or unfolded collagen molecules [26], showed high co‐localization with the ER tracer (ER‐Tracker Green), suggesting the accumulation of misfolded proteins within the endoplasmic reticulum (Figure S16C, D). Furthermore, previous studies reported that when proteasome function is impaired, the deubiquitinating enzyme USP19 mediates the secretion of misfolded proteins to the extracellular space, whereas USP19‐deficient cells lack this capability [27]. Prompted by this, we found significantly decreased proteasome markers PSMA6, PSMB1, and PSMB5 alongside upregulated USP19 expression in the bone cells of the BML group, with TRC051384 intervention restoring the expression of proteasome genes, suggesting that USP19 may mediate the secretion of misfolded proteins upon proteasome deficiency (Figure 3K,L). To clarify USP19 function, an adeno‐associated virus serotype 9 (AAV9) vector was utilized to knock down USP19 in vivo (Figure 3M), given a high targeting efficiency of AAV9 on bone cells [28]. Results showed reduced extracellular COLI accumulation after USP19 knockdown (Figure 3N) but a significant increase in cell apoptosis (Figure 3O). Moreover, western blot verified that elevated expression of key molecules in the Ras‐ERK‐c‐Fos pathway, a pro‐apoptotic pathway enriched in the bone cells of the BML group [29], which could also be inhibited by TRC051384 intervention (Figure S17), implying that the apoptosis of bone cells may be attributed to the activated Ras‐ERK‐c‐Fos pathway. Collectively, these findings demonstrate that reduced HSP70 leads to misfolded COLI accumulation, and USP19 mediates misfolded protein secretion into the extracellular matrix, ultimately driving matrix accumulation within BMLs.

### 2.4 Bone‐Derived WNT5A Promotes Expansion and Apoptosis of HTCs during BMLs Progression

**FIGURE 4:**
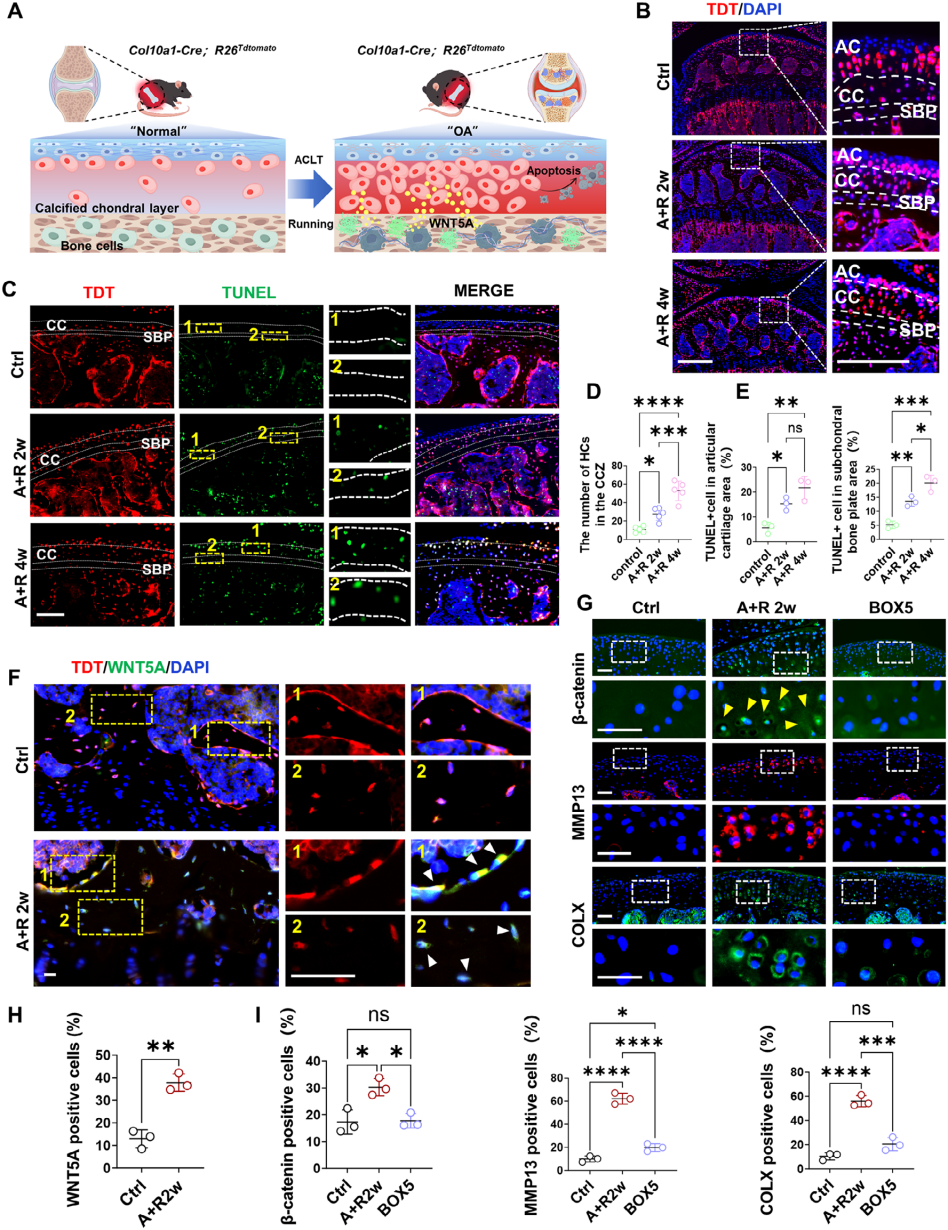
WNT5A secreted by osteoarthritic bone cells promotes chondrocyte hypertrophy and death during BML development. (A) Schematic illustration of chondrocyte hypertrophy and bone‐cartilage interactions in BML development. (B and D) Representative images of tdTomato fluorescence in the calcified cartilage of control and BML groups (B), with quantification of HTCs number in the calcified cartilage (D) (n = 5). Three white dashed lines divide the intact joint section into the cartilage region, the calcified cartilage region, the subchondral bone plate region, and the subchondral bone region. scale bars = 200 um. (C and E) Representative images (C) and quantification (E) of fluorescence staining of TUNEL of control and BML groups in the calcified cartilage layer and subchondral bone plate layer, respectively (n = 3). And the numbers 1 and 2 represent the calcified cartilage region and the subchondral bone plate region, respectively. scale bars = 100 um. (F and H) Representative images (F) and quantification (H) of immunostaining of WNT5A in the subchondral bone of control and BML groups (n = 3), with white arrows indicating WNT5A‐expressing bone cells. The numbers 1 and 2 represent the expression of WNT5A in osteoblasts and osteocytes, respectively. scale bar = 25 um. (G and I) Representative images (G) and quantification (I) of immunostaining of β‐catenin, MMP13, and COLX in the control, BML, and treatment groups (n = 3). The yellow arrows indicate β‐catenin entering nuclei in HTCs. scale bar = 25 um. Data are shown as the mean ± SD. The difference between the two groups was analyzed by Student's t‐test (H). Multiple group comparison was assessed using one‐way ANOVA (D, E, I). ^*^ *p* < 0.05, ^**^ *p* < 0.01, ^***^ *p* < 0.001, ^****^ *p* < 0.0001.

Accelerated chondrocyte hypertrophy is a hallmark of OA progression, characterized by protease secretion that degrades cartilage matrix and promotes tissue degeneration [30]. To verify the increased number of HTCs in BML samples, we employed a genetic Col10a1‐Cre mouse line to dynamically visualize HTCs during BML development (Figure 4A; Figure S18). Results demonstrated fewer HTCs in control *Col10a1‐Cre; R26^tdt+^* mice, but HTCs increased significantly as BML progressed (Figure 4B,D). TUNEL staining further revealed significantly elevated apoptosis in HTCs with synchronously exacerbated apoptosis of bone cells in the subchondral bone plate (Figure 4C,E).

ScRNA‐seq data suggest WNT5A as a core factor in promoting chondrocyte hypertrophy (Figure 2I). As a primary secretory protein in non‐canonical Wnt signaling, WNT5A not only participates in the non‐canonical Ca^2+^ pathway but also activates β‐catenin in the canonical pathway, thereby accelerating cartilage degeneration [31]. To validate the role of WNT5A in promoting chondrocyte hypertrophy, we detected WNT5A expression in bone cells of *Col10a1‐Cre; R26^tdt+^* mice. Compared to control, the WNT5A was significantly upregulated in bone cells in the BML group (Figure 4F,H). Notably, β‐catenin exhibited elevated expression and nuclear translocation in HTCs, whose activation drives cartilage matrix degradation and hypertrophy [32], consistent with our findings (Figure 4G,I; Figure S19). Additionally, administration of the WNT5A inhibitor BOX5 [33] effectively blocked cartilage degeneration. Collectively, these data demonstrate WNT5A as the key driver of chondrocyte hypertrophy during BMLs progression.

### 2.5 HSP70 Inducer TRC051384 Ameliorates OA by Inhibiting BML Formation

**FIGURE 5:**
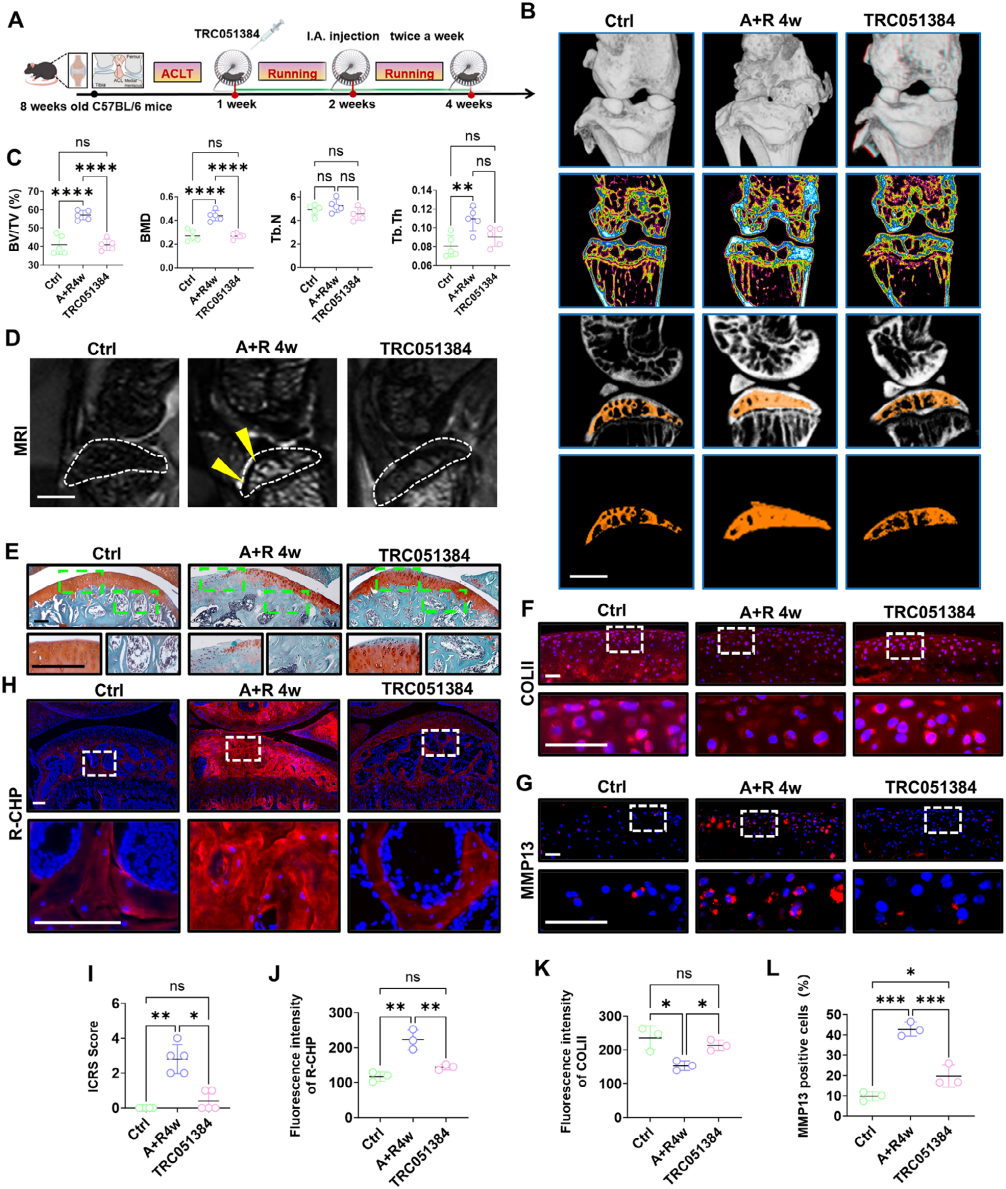
Improving HSP70 expression ameliorates OA by inhibiting BML formation. (A) Schematic diagram of drug delivery in the articular cavity of the modeling mice. (B and C) Representative µCT images (B) and quantification (C) of bone volume/trabecular bone volume ratio (BV/TV), trabecular bone number (Tb. N.), trabecular bone thickness (Tb. Th.), and bone mineral density (BMD) in the subchondral bone from each group of the mouse model (n = 5). scale bars = 1 mm. (D) Representative images of 9.4T MRI sequences of T2 phase fat suppression in each group, with yellow arrows indicating BML region and white dotted line indicating subchondral bone region. scale bars = 1 mm. (E and I) Representative images of Safranin‐O in each group of the mouse model (E) and quantification of International Cartilage Repair Society (ICRS) scores (E) (n = 5). scale bars = 50 um. (F and K) Representative images (F) and quantification (K) of immunostaining of COLII in cartilage of control, BML, and treatment groups (n = 3). scale bars = 25 um. (G and L) Representative images (G) and quantification (L) of immunostaining of MMP13 in cartilage of control, BML, and treatment groups (n = 3). scale bars = 25 um. (H and J) Representative images (H) and quantification (J) of staining of collagen hybridization peptide (R‐CHP) in control, BML, and treatment groups (n = 3). scale bars = 50 um. Data are shown as the mean ± SD. Statistical significance was assessed using the Kruskal‐Wallis test (I) or one‐way ANOVA (C, J, K, L). ^*^ *p* < 0.05, ^**^ *p* < 0.01, ^***^ *p* < 0.001, ^****^ *p* < 0.0001.

Our results revealed that the HSP70 inducer TRC051384 reversed COLI misfolding and secretion, suggesting its potential efficacy in mitigating OA progression. To assess the therapeutic effects of TRC051384 on OA, we administered the drug intra‐articularly in mice (twice a week for three weeks) (Figure 5A). Micro‐CT assessment demonstrated an increase in subchondral bone with plate‐like structure in the A+R group, indicative of osteosclerosis, and this was ameliorated by TRC051384 (Figure 5B,C). Concurrently, MRI demonstrated BMLs presented in the A+R mice but were significantly reduced in the treatment group (Figure 5D). Histology analyses indicated that the treatment markedly delayed cartilage matrix loss and osteosclerosis (Figure 5E,I). Similarly, immunostaining of COLII and MMP13 indicated reduced matrix synthesis (Figure 5F,K) and elevated catabolic activity (Figure G, L) during OA, both counteracted by TRC051384 intervention. CHP staining showed that TRC051384 prevented extracellular accumulation of misfolded collagen (Figure 5H,J). Collectively, these data demonstrate the robust efficacy of TRC051384 in alleviating OA pathology.

### 2.6 Proteasome Gene Expression Impacts the Risk of Developing OA and Decreases in Osteoarthritic Subchondral Bone

**FIGURE 6:**
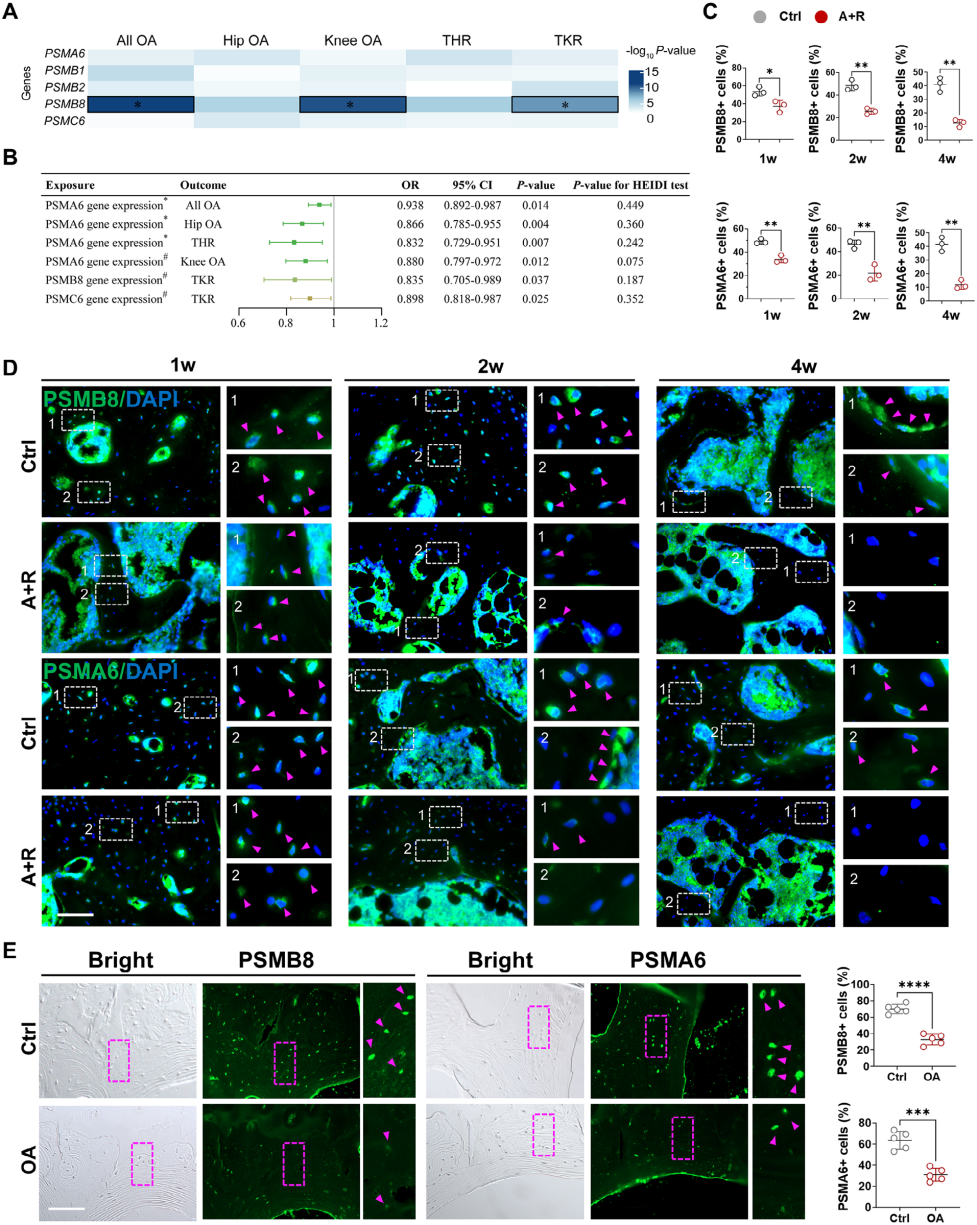
Decreased proteasome gene expression and impaired function are linked to the risk and development of OA. (A) Genome‐wide association study (GWAS) meta‐analysis of the association of genes with OA‐related traits. ^*^ P‐value < 5×10^−8^. (B) Mendelian randomization analysis of the association between the expression levels of PSMB8, PSMA6, and PSMC6 in whole blood and the risk of OA. ^*^ Gene expression was obtained from whole blood tissue based on the eQTLGen Consortium. # gene expression was obtained from whole blood tissue based on the GTEx project. (C, D) Representative images (C) and quantification (D) of immunostaining of PSMB8 and PSMA6 in control and BML groups (n = 3), with purple arrows indicating PSMA6‐ and PSMB8‐positive bone cells. scale bars = 50 um. (E) Representative images and quantification of immunostaining of PSMB8 and PSMA6 in subchondral bone samples from regions underlying damaged and intact cartilage of OA patients (n = 5), with purple arrows indicating PSMA6‐ and PSMB8‐positive bone cells. scale bars = 100 um. Data are shown as the mean ± SD. The difference between the two groups was analyzed by Student's t‐test (C, E). ^*^ *p* < 0.05, ^**^ *p* < 0.01, ^***^ *p* < 0.001, ^****^ *p* < 0.0001.

Since proteasome dysfunction contributes to aberrant secretion of misfolded collagen, we first set out to evaluate whether expressions of proteasome‐related genes could impact OA risk and development by analyzing human GWAS meta‐analysis data (N = 1,962,069) [34]. The results showed that variants within the PSMB8 gene region (±250 kb) demonstrated genome‐wide significant associations with multiple OA phenotypes, including all OA (lead SNP rs2021408, P‐valueGWAS = 4.71×10‐12), knee OA (rs1431402, P‐valueGWAS = 1.78×10‐11), and total knee replacement (rs3130169, P‐valueGWAS = 5.00×10‐9) (Figure 6A). Additionally, suggestive associations were observed for variants near the loci of multiple proteasome genes, PSMA6, PSMB1, PSMB2, and PSMC6 (P‐valueGWAS < 5×10‐4) (Figure 6A). Mendelian randomization analyses demonstrated that genetically predicted increased expression levels of PSMB8, PSMA6, and PSMC6 in whole blood tissue were associated with decreased risk of OA (P‐valueMR < 0.05) (Figure 6B). Prompted by these findings, we further validated the expression of PSMB8 and PSMA6 in mouse and human subchondral bone. Immunostaining results showed that in BML mice, expression levels of both genes began to decline at one week post‐modeling and continued to decrease progressively with prolonged modeling time (Figure 6C,D). Concurrently, analysis of the subchondral bone samples corresponding to the intact cartilage regions and damaged cartilage regions identified by histological scoring revealed significantly reduced expression levels of PSMB8 and PSMA6 in the subchondral bone beneath the damaged cartilage areas compared to the intact regions (Figure 6E; Figure S20). Collectively, these findings established a link between decreased expression of proteasome genes, indicative of impaired proteasome function, and the risk and development of OA.

## 3 Discussion

In this study, we established a stable mouse BML model and employed scRNA‐seq combined with genetic lineage tracing techniques to decipher the previously unknown pathological phenotype and pathogenesis of early‐stage BMLs. We propose that COLI misfolding resulting from reduced HSP70 expression in bone cells and USP19‐mediated extracellular secretion mechanisms constitute crucial factors contributing to pathological bone matrix accumulation within BMLs. Concurrently, using *Col10a1‐Cre; R26^tdt+^* tracing mice, we found a sequential increase in HTCs followed by apoptosis, and we also uncovered that bone‐derived WNT5A is the principal driver of chondrocyte hypertrophy. Intra‐articular administration of the HSP70 inducer TRC051384 effectively attenuated BML formation. These findings deepen our understanding and provide novel insights and therapeutic targets for early BML pathogenesis.

In recent years, the intervention strategy for OA has gradually shifted from ‘symptom relief’ to ‘early intervention’, necessitating a thorough understanding of pathological features across OA stages, particularly the reversible damage phase [1, 35]. Subchondral bone damage precedes cartilage degeneration, potentially due to the higher mechanical force sensitivity of osteo‐lineage cells compared to chondrocytes. Ranging from the principle of Wolff's law at the macroscopic level to the efficiency of mechanosensing at the cellular level, osteo‐lineage cells could rapidly respond to changes in stress and initiate remodeling processes, while chondrocytes, restricted by their buffering matrix and low metabolic state, may exhibit relatively delayed responses to mechanical stimuli [36, 37]. However, the current clinical field faces a dual bottleneck: the absence of sensitive early diagnostic biomarkers and effective interventions, which leads to most patients being in an irreversible late stage when they seek treatment, ultimately requiring joint replacement. Among existing imaging techniques, X‐ray/CT are limited to detecting late‐stage structural lesions, while MRI can identify BMLs at earlier stages [38]; yet the clinical significance of early‐stage BML remains underappreciated, with current treatment still relying on non‐steroidal anti‐inflammatory drugs (NSAIDs) for symptomatic relief [35]. Likewise, BML‐related basic research is constrained by the lack of early human samples and effective animal models. Previously reported animal models have some limitations, including random lesion occurrence, irregular time windows, and low incidence rates [8, 39]. The ACLT model is recognized as an ideal OA model due to its precise simulation of joint mechanical misalignment, which induces aberrant load distribution and shear forces. This joint instability directly drives subchondral bone remodeling and progressive articular cartilage degeneration [40]. However, in our initial studies, ACLT surgery alone failed to stably induce BML phenotypes. A critical limiting factor was the sharp reduction in mice's postoperative activity (limited to localized movement during feeding and drinking), resulting in insufficient aberrant stress stimulation. A breakthrough came with the introduction of forced exercise, where controlled forced running wheel exercise not only induced stable BML occurrence but also showed diffusion expansion related to exercise duration. It is worth noting that in the OA mouse model used in this study, BMLs specifically occur in the tibial plateau. One of the possible explanations is that the load distribution associated with quadrupedal locomotion and the distinct anatomical angulation of the mouse knee joint collectively concentrate abnormal mechanical stress on the more directly loaded subchondral bone of the tibia [41]. Building on this, we developed a synergistic modeling strategy that combines ACLT surgery with controlled forced running wheel exercise, achieving the establishment of a stable early‐stage BML animal model. Our model delivers critical reference value in terms of early BML phenotypes, mechanisms, and interventions: (i) establishing precise characterization of early BML pathological phenotypes and defining research baselines; (ii) elucidating core mechanisms through which BMLs drive OA progression; and (iii) providing a critical theoretical foundation for implementing precise interventions during the initiation window of BML to prevent OA progression.

Single‐cell sequencing revealed no significant differences in the subtypes or quantities of bone cell subpopulations between the two groups, leading us to hypothesize bone‐cell dysfunction in the BML group. Further studies have demonstrated that the OA bone cells were significantly impaired in heat shock protein family activity and protein folding capacity, principally the HSP70 family. Beyond its recognized role in protein folding [42], HSP70 also exerts broad cytoprotective effects by inhibiting apoptotic signaling pathways [43] and modulating autophagy processes [44], which represent potential downstream mechanisms of HSP70. Critically, protein folding relies on molecular chaperone guidance to prevent erroneous conformations [20], and its fidelity is precisely regulated by the chemical microenvironment and mechanical stress. When aberrant stress disrupts folding topology [45] and energy landscapes [46], it triggers misfolded protein aggregates, subsequently inducing endoplasmic reticulum stress and loss of protein function [25, 47]. Under physiological conditions, approximately 30% of nascent proteins form misfolded conformations due to translational errors, sequence aberrancies, or defective modifications [48]. And the cell could maintain homeostasis through molecular chaperone refolding and proteasomal degradation. Once homeostasis is disrupted, particularly when HSP70 dysfunction leads to the failure of its protective mechanisms, it not only causes protein folding abnormalities but also impairs stress adaptation and accelerates the systemic collapse of cellular homeostasis. [47, 49]. Both this study and previous work collectively indicate that targeting HSP70 represents an effective strategy for restoring cellular homeostasis and intervening in related disease processes. Our study identified concurrent loss of protein folding function and proteasome dysfunction in bone cells within the BML region. This dual impairment not only promotes excessive misfolded protein accumulation but also obstructs their clearance via proteasomal degradation. Ultimately, accumulated misfolded proteins undergo USP19‐mediated extracellular secretion [27], depositing within the ECM and directly driving a proteinopathy characterized by misfolding‐induced pathological bone matrix accumulation. The USP19‐mediated secretion of misfolded proteins represents a transient survival strategy under proteotoxic stress, temporarily alleviating the intracellular burden and delaying apoptosis [50]. However, the extracellular accumulation of these proteins promotes the formation of persistent aggregates [51], which disrupt bone matrix integrity and exacerbate chronic inflammation [52]. Consequently, while USP19 activity supports immediate cell survival, it inadvertently shifts the proteostasis crisis from the intracellular to the extracellular compartment, thereby driving the formation of BML. This dualistic role underscores how a compensatory cellular response can evolve into a key driver of chronic tissue degeneration.

The subchondral bone and cartilage collectively constitute an osteochondral unit adapted for load transmission [53]. Although early pathological changes may target a single tissue, close biological and physical interactions ultimately affect the entire unit [5, 54]. In our study, the osteochondral interaction revealed that the WNT5A ligand in bone cells most strongly correlated with the FZD4/5‐LRP5 receptor in HTCs, with a 24‐fold increase in HTCs number, implying that bone‐derived WNT5A may promote chondrocyte hypertrophy. WNT5A, a core ligand of the non‐canonical WNT pathway, regulates cartilage formation during embryonic development by promoting chondrocyte differentiation while inhibiting maturation [55]; and its expression is significantly elevated in OA tissues [56, 57]. As a secreted protein, WNT5A activates both canonical and non‐canonical pathways via ROR2/FZD receptors, specifically through the Ca^2^^+^/calmodulin‐dependent protein kinase II (CaMKII)‐RUNX2 axis and β‐catenin nuclear translocation [31, 58]. This dual activation synergistically upregulates COLX and MMP13 expression, thereby accelerating cartilage matrix degradation and inducing chondrocyte hypertrophy [59]. Given that chondrocyte hypertrophy is a hallmark of OA progression, our study employed *Col10a1‐Cre; R26^tdt+^* mice to achieve the first visualization of bone‐derived WNT5A‐driven chondrocyte hypertrophy, providing direct evidence for osteochondral crosstalk and the hypertrophy process. Meanwhile, we discovered that the sequential increase of HTCs in OA progression mainly tended to apoptosis, revealing the terminal fate of HTCs in OA. However, the mechanistic link between the secretion of WNT5A and the dysfunction of HSP70 warrants future studies. Nevertheless, our data show that inhibiting HSP70 in osteo‐lineage cells significantly increases WNT5A secretion, suggesting an HSP70‐dependent secretion of WNT5A. Consistently, it has been reported that HSP70 deficiency may induce oxidative stress to activate AP‐1/NF‐κB and thereby upregulate WNT5A transcription [60]; moreover, it could trigger ERS and the unfolded protein response (UPR), leading to XBP1s‐mediated WNT5A upregulation [61].

The core translational significance of this study lies in using TRC051384 as a pharmacological tool to validate for the first time the feasibility of the ‘targeted enhancement of HSP70 function’ strategy in reversing BML phenotypes. TRC051384 is a small‐molecule HSP70 activator that has previously been primarily focused on neurological disorders [22], with its protective effect closely associated with HSP70‐mediated protein quality control mechanisms. From a molecular mechanism perspective, its action may involve the specific activation of HSF1 transcriptional activity, thereby enhancing the overall efficiency of the protein folding, repair, and degradation network [62]. Our study applied TRC051384 to a skeletal system disease model, not only expanding its potential clinical application scenarios but also mechanistically confirming the critical role of HSP70 in maintaining proteostasis in osteo‐lineage cells. The pleiotropic nature of TRC051384 could exhibit dual characteristics in complex pathological environments: on the one hand, its non‐specific modulation of other protective stress pathways may produce synergistic effects, enhancing overall cytoprotection, which may partly explain its significant efficacy in the BML model; on the other hand, this broad activity may also introduce off‐target risks, representing an area for optimization in future therapeutic development.

Several limitations exist. First, while the established mice A+R model successfully recapitulates BMLs pathology, resolution limitations necessitate future validation in large animals such as rabbits or pigs. Second, although HSP70 overexpression effectively prevents early OA progression, the precise regulatory mechanism linking reduced HSP70 expression and aberrant mechanical stress requires further elucidation. Lastly, the availability of early human OA clinical samples remains limited. Overall, this study successfully established a mouse model of BMLs and constructed a complete scRNA‐seq atlas, revealing the mechanisms underlying BML formation, which not only fills the research gap of BMLs in early OA, but also provides therapeutic targets for early OA diagnosis and intervention.

## 4 Experimental Section

### 4.1 Study Design

The objectives of the present study were to investigate the pathological characteristics and molecular mechanisms of BMLs and to develop and validate drugable targets for early OA featuring on BMLs. Using serial MRI scans of an individual from the OAI cohort and a standardized mouse model combining ACLT with forced wheel running, we confirmed a significant correlation between BML development and cartilage degeneration. Through the establishment of a single‐cell atlas of murine BMLs combined with molecular biology assays and physical characterization techniques, we demonstrated that disrupted expression of HSP70 and USP19 in osteoarthritic bone cells drives BML pathology characterized by proteasome dysfunction and aberrant secretion of misfolded collagen. WNT5A secreted by these osteoarthritic subchondral osteoblasts was found to accelerate HTC hypertrophy and death through gene lineage tracing technology. We also evaluated the therapeutic efficacy of the HSP70 inducer TRC051384 against BML formation and OA progression. Based on our prior experience with comparable experimental models and projected average variability levels, we determined cohort sizes and statistical power. For repeated experimental analyses, a minimum of three biological replicates per group were utilized. No animal specimens or outliers were excluded from any analyses. Mice were randomly assigned to groups, and observers were blinded to the experimental conditions during analysis. Detailed sample sizes, biological replicates, and statistical methodologies are provided in the respective figure legends.

### 4.2 Human Samples

This study was approved by the Ethics Committee of Xijing Hospital, Fourth Military Medical University (Approval Number: KY20232024‐F‐1) and received informed consent from each patient. Human subchondral bone samples were collected from five patients with OA diagnosed and undergoing total knee arthroplasty (TKA). Based on the ICRS cartilage scoring criteria, subchondral bone beneath damaged cartilage was categorized as the ‘damage area’, while subchondral bone beneath relatively healthy cartilage was categorized as the ‘intact area’. Immediately after collection, all specimens were rapidly frozen in liquid nitrogen and maintained at ‐80°C until further processing. Table S2 contains detailed demographic and clinical characteristics of the patients.

### 4.3 Mice

The *Col10a1⁃Cre* mice were kindly provided by Professor Kathryn S. E.Cheah from the School of Biomedical Sciences, The University of Hong Kong. *ROSA‐Tdtomato* mice were purchased from Jackson Laboratory (Bar Harbor, Maine, USA; Strain NO. 007914). To enable in vivo tracking of hypertrophic chondrocytes (HTCs, Col10a1^+^), *Col10a1⁃Cre* mice were crossed with *ROSA‐Tdtomato* mice to generate *Col10a1‐Cre; R26^tdt/+^* offspring. All other experimentally relevant animals were wild‐type C57BL/6 background mice. And all animals were housed under specific pathogen‐free (SPF) conditions at the Experimental Animal Center of the Fourth Military Medical University. Mice were euthanized via cervical dislocation under isoflurane anesthesia. All animal procedures strictly adhered to the Fourth Military Medical University Guidelines for Animal Welfare and Ethics (Approval Number: IACUC‐20230023).

### 4.4 Statistical Analysis

Statistical analysis was performed with GraphPad Prism version 9.0, and the results were given as mean ± standard deviation (SD) from ≥3 independent experiments. The normality of the data was confirmed by the Shapiro‐Wilk normality test. Between‐group comparisons employed a two‐tailed Student's t test, Kruskal‐Wallis test, one‐way ANOVA or two‐way ANOVA when appropriate and as indicated in figure legends. ^*^ *P* < 0.05, ^**^ *P* < 0.01, ^***^ *P* < 0.001 and ^****^ *P* < 0.0001, ns was no significance with *P* > 0.05.

## Author Contributions

L.Y., C.Z., and H.X. conceived and designed the study. C.Z. and L.Y. secured funding for the work. The methodology was developed by H.X., T.H., Y.Q., J.L., B.N., X.X., H.W., P.W., and G.C. Experimental execution, data analysis, and interpretation were performed by H.X., T.H., Y.Q., B.N., X.X., P.W., S.Y., G.D., R.W., and Z.G. Visualization was implemented by H.X., Y.Q., J.L., R.W., and C.Z. Supervision was implemented by W.L., Z.M., H.Z., Z.Z., Z.L., Q.M., C.Z., and L.Y. The initial manuscript was drafted by H.X., C.Z., and L.Y. All authors critically revised the manuscript and approved the final version.

## Funding

National Natural Science Foundation of China 82130070 (LY). National Natural Science Foundation of China 82394442 (LY). National Natural Science Foundation of China 82422043 (CZ). Shaanxi Province Health Science and Technology Innovation Capability Enhancement Program 2025TD‐14 (LY).

## Conflicts of Interest

The authors declare no conflicts of interest.

## Supporting information




**Supporting File**: advs74417‐sup‐0001‐SuppMat.docx.

## Data Availability

The data that support the findings of this study are available from the corresponding author upon reasonable request.
