# Supplementary material for: Single‐Cell Atlas of Subchondral Bone Marrow Lesions Reveals Proteostasis Dysfunction as a Druggable Mechanism for Early Osteoarthritis
Source: Adv Sci (Weinh). 2026 Feb 13;13(26):e16720. doi: 10.1002/advs.202516720 (PMC13159136; doi:10.1002/advs.202516720)
Supplement: Supplementary file 1 — Supporting File: advs74417‐sup‐0001‐SuppMat.docx. [file ADVS-13-e16720-s001.docx]

**Supplementary Materials**

**Single-cell Atlas of Subchondral Bone Marrow Lesions Reveals Proteostasis Dysfunction as a Druggable Mechanism for Early Osteoarthritis**

**Authors:** Hailun Xu^1,2^, Ting He^1^, Yu Qian^3,4^, Jia Li^5^, Bowei Ni^1^, Xiaojie Xu^1^, Huanbo Wang^1,6^, Peng Wang^1,2^, Guoqing Cao^1,7^, Siqi Ying^1^, Guangyu Ding^1^, Rong Wang^5^, Zenghui Gu^3,4^, Wei Li^2^, Zhentao Man^2^, Houfeng Zheng^3,4^, Zhaohua Zhu^8,9^, Zhen Li^10^, Qingjun Meng^11^, Chao Zheng^1^*, Liu Yang^1,12^*

**Affiliations:**

^1^Institute of Orthopedic Surgery, Xijing Hospital, Fourth Military Medical University; Xi’an, 710032, China

^2^Shandong Provincial Hospital Affiliated to Shandong First Medical University; Jinan, Shandong Province 250021, P. R. China

^3^Suzhou Laboratory of Precision Health and Data Science, the Second Affiliated Hospital of Soochow University, Suzhou, Jiangsu, China

^4^Institute of Health Data Science, Soochow University, Suzhou, Jiangsu, China

^5^Division of Orthopaedic Surgery, Department of Orthopedics, Nanfang Hospital, Southern Medical University; Guangzhou, Guangdong, China

^6^Department of Orthopedics, Air Force Hospital of Eastern Theater Command; Nanjing, Jiangsu, China

^7^The 989th Hospital of the Joint Logistics Support Force of the Chinese People's Liberation Army; Luoyang, Henan, 471000, China

^8^Clinical Research Centre, Zhujiang Hospital, Southern Medical University; Guangzhou, Guangdong, China

^9^Department of Rheumatology, Royal North Shore Hospital and Sydney Musculoskeletal Health, Kolling Institute, University of Sydney; Sydney, Australia

^10^AO Research Institute Davos, 7270; Davos Platz, Switzerland

^11^Faculty of Biology, Medicine and Health, University of Manchester; Oxford Road, Manchester, M13 9PT, UK

^12^Medical Research Institute, Northwestern Polytechnical University; Xi’an, 710072, China

* Correspondence to

Liu Yang, Ph.D., Institute of Orthopedic Surgery, Xijing Hospital, Fourth Military Medical University, Xi’an, People’s Republic of China.

E-mail: yangliu@fmmu.edu.cn. Tel: 86-29-84775291

Chao Zheng, Ph.D., Institute of Orthopedic Surgery, Xijing Hospital, Fourth Military

Medical University, Xi’an, People’s Republic of China.

E-mail: slc26a2@hotmail.com. Tel: 86-29-84775291

**The PDF file includes:**

Experimental Section

Figures. S1 to S20

Table S1-S3

**Experimental Section**

**Construction of the BML mouse model**

Twelve-week-old male C57BL/6 mice were subjected to ACLT surgery under isoflurane anaesthesia in a sterile environment. Specifically, the knee joint was exposed microscopically through a medial parapatellar incision and the ACL was severed using microsurgical scissors. Sham-operated controls had only the joint exposed without ligamentous injury. Subsequently, forced running wheel exercise was performed with an electronically controlled motorised wheel drive device (Lafayette Instrument Company, USA) with up to 20 exercise wheels, each capable of holding one mouse. Running wheel exercise was initiated 1 week after surgery, initially running at 5 m/min for 15 min to acclimatise to the rhythm, and then switching to 7 m/min for 45 min for 1 h per day, 5 days per week^[1]^. Mice were randomly divided into four groups: (i) control group (sham operation), (ii) ACLT group, (iii) control + running group (C+R), and (iv) ACLT + running group (A+R).

**Single-cell Dissociation**

For each group containing a total of 10 12-week-old male C57BL/6 mice, tibial plateau and distal femoral condyle tissues without growth plates were isolated from both hind limb metaphyses under a microscope. The tissues were removed and kept in MACS Tissue Storage Solution (Miltenyi Biotec) until processing. The tissue samples were processed as described below. Briefly, samples were first washed with phosphate-buffered saline (PBS), minced into small pieces (approximately 1mm^3^) on ice and enzymatically digested with 2 mg/mL Collagenase II, 1 mg/mL Dispase, DNase I 30 U/µL(Worthington) for 60 min at 37 °C, the supernatant was added to an equal volume of 1640 culture solution to terminate digestion and sieved through a 40 µm cell strainer and centrifuged at 400 g for 6 min. After the supernatant was removed, the pelleted cells were suspended in red blood cell lysis buffer (Miltenyi Biotec) to lyse red blood cells. After washing, for MACS sorting: cell pellets were re-suspended into 80 μL PBS containing 0.5% BSA, incubated with 20 μL Anti-CD45 microbeads on ice for 15 min. After that, samples were diluted by 500 μL buffer, and the negative cells were collected. The cell pellets were re-suspended and stained with AO（Acridine Orange）/PI（Propidium Iodide）for viability assessment using Countstar Fluorescence Cell Analyzer.

**Single-cell RNA Sequencing**

The scRNA-Seq libraries were generated using the 10X Genomics Chromium Controller Instrument and Chromium Single Cell 3’ V3 Reagent Kits (10X Genomics, Pleasanton, CA). Briefly, cells were concentrated to approximately 1000 cells/µL and loaded into each channel to generate single-cell Gel Bead-In-Emulsions (GEMs). After the RT step, GEMs were broken and barcoded cDNA was purified and amplified. The amplified barcoded cDNA was fragmented, A-tailed, ligated with adaptors and index PCR amplified. The final libraries were quantified using the Qubit High Sensitivity DNA assay (Thermo Fisher Scientific) and the size distribution of the libraries were determined using a High Sensitivity DNA chip on a Bioanalyzer 2200 (Agilent). All libraries were sequenced by DNBSEQ-T7 Sequencer (MGI, Shenzhen, China) on a 150 bp paired-end run.

**Immunofluorescence and Histology**

The mice knee joints were quickly removed and rinsed with pre-cooled PBS and subsequently fixed in 4% paraformaldehyde for 24 h. The tissues were then decalcified for 2 weeks after which they were partially dehydrated and embedded in paraffin wax for paraffin sectioning, and partially placed in a 30% sucrose solution before being frozen for sectioning. Sagittal sections 6-μm thick were cut from the medial aspect of murine knee joints, and for immunofluorescence, sections were first repaired with sodium citrate acidic repair solution (Beyotime, P0081) at 80° C for 10 min, followed by sealing with 2% BSA (Beyotime, P0252) for 1 h. Sections were then incubated with primary antibody for 16 h at 4° C, washed three times with PBS at incubated with a 1:1000 dilution of Alexa Fluor 488 or 594 for 1 h at room temperature. Sections were then blocked with DAPI-containing blocking solution. The sections were stained with Safranin O Fast Green (SO&FG), hematoxylin-eosin (H&E), Tartrate resistant acid phosphatase (TRAP) and Sirius red stain, and histopathological changes in articular cartilage and subchondral bone were assessed using the International Cartilage Repair Society (ICRS) ^[2]^ and Osteoarthritis Research Society International (OARSI) scoring system ^[3]^. Also, to enhance the comparability and statistical power of the experimental data and to further investigate the spatiotemporal relationship and pathological mechanisms between cartilage degeneration and the development of BML, we selected the tibial plateau, the primary site of BML occurrence, as the unified region for analysis.

**Magnetic resonance imaging**

High-resolution MRI of the mouse knee joint was performed using a 9.4T Bruker BioSpec system equipped with a circular surface coil for optimal signal reception. Mice were anesthetized with 1.5-2% isoflurane in oxygen and carefully positioned to ensure close contact between the knee joint and the surface coil. Coronal T2-weighted images were acquired using a TurboRARE sequence with the following parameters: TR = 2500 ms, TE = 24.8 msslice thickness = 0.6 mm. And we selected the fat suppression option in the operating interface. The surface coil was carefully positioned and secured to maximize signal-to-noise ratio while minimizing motion artifacts.

**Micro-computed tomography**

Knee tissues were fixed with 4% paraformaldehyde (4° C, 24 h) and were scanned using a Bruker Skyscan 1276 system (Bruker MicroCT, Germany) at 60 kV voltage, 200 μA current, and 580 ms integration time, with a CS intrinsic rotation of 180.0°. To assess the total volume of tibial subchondral bone in each knee and to generate transverse and sagittal reconstruction images, the region of interest (ROI) in each two-dimensional (2D) section was manually outlined, followed by three-dimensional reconstruction and volume calculations using CTVol software (version v2.3.3.0). Various bone microstructural parameters were analyzed and statistically calculated, including bone volume fraction (BV/TV), bone mineral density (BMD), trabecular number (Tb.N), and trabecular thickness (Tb.Th). To ensure objectivity, sample identifiers were blinded by random coding by independent researchers prior to analysis.

**Extraction method of subchondral bone tissue in mice**

The operation was completed in a cellular ultra-clean bench. Microscopically, the cartilage-bone composite tissue above the growth plate obtained by blunt dissection is placed in pre-cooled PBS and kept moist. The target area could be exposed by carefully trimming the surrounding excess fat or connective tissue with microscopic scissors. Prepare 0.1% type II collagenase solution (Bioshrap, BS164), place the composite tissue in the enzyme solution and incubate at 37° C for 10-15 min, removing it every 5 min and observing the degree of cartilage softening and the edges of the cartilage for buckling under the microscope. The digestion was terminated by adding pre-cooled EDTA-containing buffer and left on ice for 5 min. Finally, the tissue was transferred to a PBS-containing petri dish, and under the microscope, the edges of the cartilage were grasped with microscopic forceps and gently lifted along the junction with the bone; however, if the cartilage was not loosened, it should not be forcibly peeled off; instead, a microscopic spatula should be used to gently scratch from the interface, taking advantage of the reduced adhesion of the cartilage after enzyme digestion to peel off the cartilage in layers. Finally, a more complete subchondral bone tissue was obtained.

**Western Blotting**

The subchondral bone tissues of mouse knee joints under different treatments were surgically obtained, and protein extraction was performed according to the experimental steps of the protein extraction kit, and the total protein concentration was determined by the BCA kit (Beyotime, P0012). The extracted proteins were mixed with the loading buffer and boiled at 100°C for 10 min in a metal bath. Samples (30 μg/lane) were separated by gel electrophoresis through a 10% polyacrylamide precast gel and using Tris-Glycine electrophoresis buffer system and transferred to a PVDF membrane (Merck, IPFL00010). After being blocked with 5% skimmed milk on a shaker for 1 h at room temperature, the membrane was incubated with primary antibody overnight at 4° C. The membrane was then incubated with secondary antibody for 1 hour at 37° C on a shaker. Imaging was developed using an enhanced chemiluminescence (ECL) imaging kit (Thermo Fisher Scientific).

**Extracellular matrix production from subchondral bone tissue**

As previously described^[4]^, in brief, the blood was washed with pre-cooled PBS immediately after sampling, followed immediately by decellularisation. As follows, the decellularization buffer was first prepared, consisting of 1% TritonX-100 (Solarbio, T8200), 0.1% NH4OH (Aladdin, A299570), 1 mM protease inhibitor (MedChemExpress, HY-K0010), and then immersed for 24 hours at 4° C, with fresh buffer replacement every 12 hours. It was then incubated with DNase I (50 U/mL) for 2 h at 37° C to degrade residual nucleic acid fragments. The supernatant was subsequently removed by centrifugation (4° C, 10,000 g, 15 min), rinsed with PBS and centrifuged again to retain the precipitated **extracellular matrix (**ECM).The remaining precipitate was subjected to protein extraction as described in the western blot procedure above.

**Co-staining of ER-Tracker and CHP collagen hybridization probe**

As mentioned previously, paraffin sections were deparaffinised and rinsed 3 times with PBS. R-CHP (3Helix) was diluted with PBS to a working solution concentration of 20uM, and when prepared, it was quickly heated in a metal bath at 80° C for 5 min, followed immediately by a rapid cooling on ice for 15-30 s, and then quickly added to the tissues, and incubated at 4° C for 2 h. After 3 times of PBS rinsing, the prepared solution at a concentration of 1 uM of ER TrackerTM Green (BODIPY™ FL, E34251), and the solvent was DMSO (Solarbio, D8371). After incubation at 37° C for 30 min and 3 washes in PBS, the slices were blocked with a DAPI-containing blocking solution, and finally visualised with a confocal microscope (Zeiss, Jena, Germany).

**TEM observation**

For transmission electron microscopy (TEM) characterization, sagittal knee joint specimens were sequentially processed through graded tissue preparation. Initial fixation was performed in 3% glutaraldehyde (0.1 M phosphate buffer, pH 7.4) for 24 h at 4° C, followed by secondary fixation using 1% osmium tetroxide under identical conditions. Subsequent dehydration involved an ascending acetone gradient (30% to absolute) prior to infiltration with pure EMBed 812 epoxy resin. Following thermal polymerization, semithin sections (500 nm) were mounted on glass slides and counterstained with 1% methylene blue for preliminary evaluation. Ultrathin sections (70-90 nm) prepared with a Leica UC7 ultramicrotome underwent dual-contrast staining with 2% Uranyl acetate and sodium citrate solutions. Imaging analysis was ultimately conducted on a field-emission TEM system (HT7800/HT7700, hitachi, Tokyo, Japan) operating at 100 kV acceleration voltage.

**Picrosirius Red**

First, the frozen sections were taken out from -20° C refrigerator to return to room temperature, fixed with 4% Paraformaldehyde fixative for 15 min and then rinsed with running water. Then, the sections were immersed in Picrosirius Red A (Servicebio, G1078), and placed in an oven at 65° C for 30 min; the sections were slightly shaken out of the water and immersed in modified Picrosirius Red B for 2 min; rinsed slightly in water, and then immersed in modified Picrosirius Red C for 30 min; then the sections were rinsed in water, and then in turn, put into three cylinders of anhydrous ethanol each for 3-5 s for rapid rinsing to dehydrate the slices, then put into clean xylene for 5 min and sealed with neutral gum. The slices were then washed with water and dehydrated by rinsing in three cylinders of anhydrous ethanol for 3-5 s each. Finally, the sections were observed by microscope (Nikon Eclipse E100, Japan), and the images were captured and analyzed.

**Calcein double labeling**

Calcein double labelling was used to detect the speed of bone formation. Prepared calcineurin solutions (0.15 mol/L NaCl and 2% NaHCO_3_) were injected intraperitoneally (10 mg/kg body weight) on days 14 and 2 prior to harvesting mouse knee bone samples. Dynamic histomorphometric indices of bone formation, including mineral attachment rate, mineralised surface and bone formation rate, were calculated as previously described^[5]^.

**In vivo drug administration**

To observe the therapeutic effect of HSP70 inducer TRC051384 on A+R model mice, the researchers injected TRC051384 (purchased from Proteintech) into the articular cavity of the knee joints in mice from the beginning of the second week to the end of the fourth week after the operation, and the dosage was used as 2 mg/kg, that is, the concentration of the drug was 5mg/mL, and the volume of each injection was 10uL, and the average body weight of each mouse was 25 g. The control solvents were prepared from 2% DMSO, 30% PEG 300, 2% Tween 80, ddH_2_O. The drug was administered twice a week for 3 weeks.

**Association Analysis of Candidate Genes with Osteoarthritis Risk**

We evaluated the association of candidate genes with osteoarthritis (OA) risk using published GWAS summary statistics for five OA phenotypes: all OA, hip OA, knee OA, total hip replacement (THR), and total knee replacement (TKR)^[6]^. For each gene, variants within ±250 kb of its coding region were extracted and tested for association with OA. Genome-wide significance and suggestive associations were defined as P < 5×10⁻⁸ and 5×10⁻⁸ ≤ *P* < 5×10⁻⁴, respectively^[7]^. Additionally, we performed summary-based Mendelian Randomization (SMR) to assess causal effects of candidate gene expression on OA as previously reported^[8]^. Briefly, Cis-eQTLs (*P* < 5×10⁻⁸ in relevant tissue) served as instrumental variables, and linkage clumping followed default SMR protocols (--ld-upper-limit 0.9 --ld-lower-limit 0.05 --peqtl-heidi 1.57e-3 --heidi-min-m 3 --heidi-max-m 20 --cis-wind 2000)^[8]^.


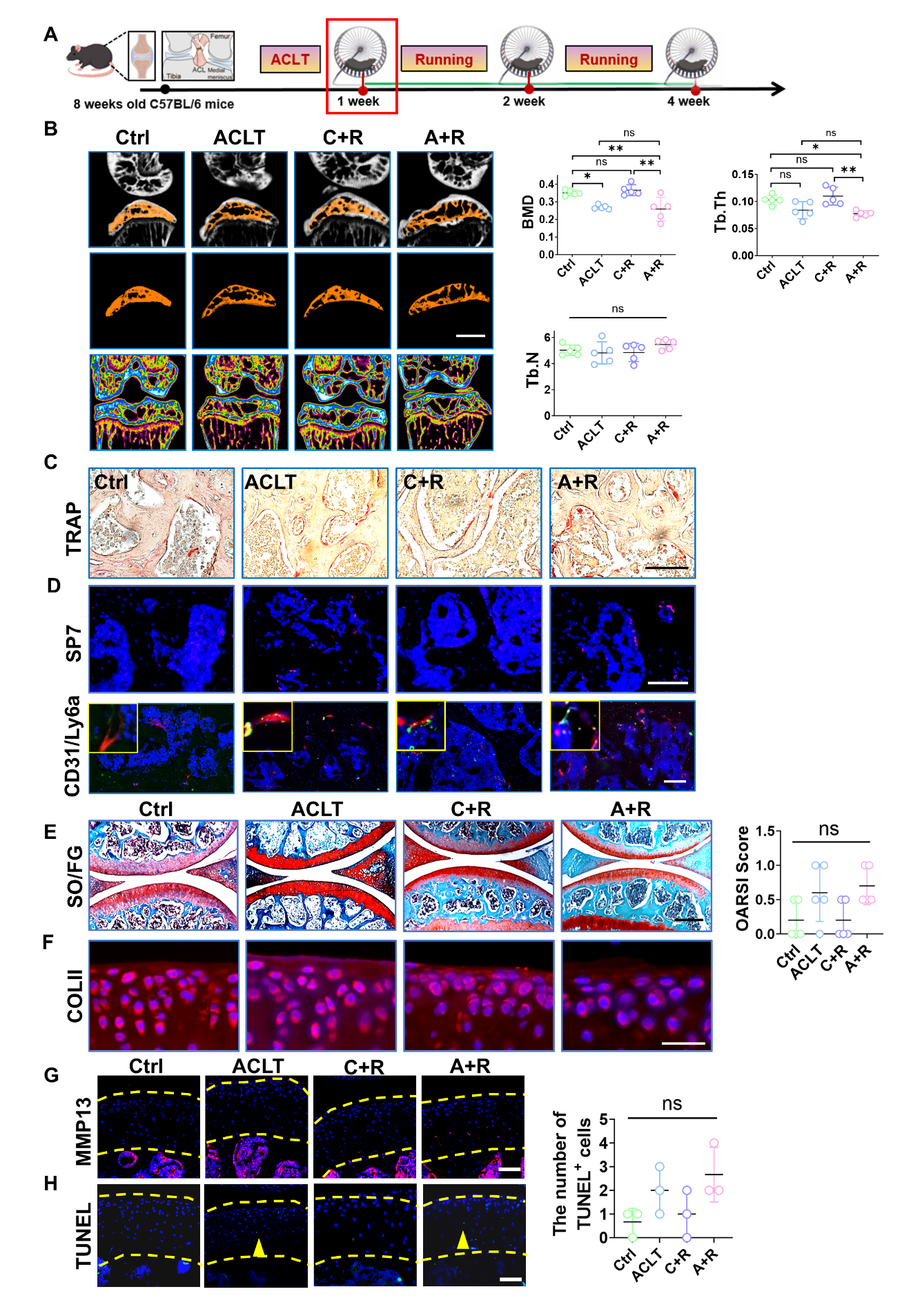


**Figure S1. Characterization of the BMLs animal model at 1 week post-modeling**

(A) Schematic diagram of constructing the combined anterior cruciate ligament transection (ACLT) and forced wheel running model (A+R) at one week post-modeling.

(B) Representative μCT images and quantification of the trabecular bone number (Tb. N.), trabecular bone thickness (Tb. Th.), and bone mineral density (BMD) in the subchondral bone of tibia from each group of the mouse model (n = 5). scale bars = 1 mm.

(C) Representative images of staining of tartrate resistant acid phosphatase (TRAP) in each group of the mouse model (n = 5). scale bars =50 um.

(D) Representative images of immunostaining of SP7 and CD31/Ly6a in each group of the mouse model (n = 3). scale bars =50 um.

(E) Representative images of Safranin-O in each group of the mouse model and quantification of OARSI scores (n = 5). scale bars =200 um.

(F) Representative images of immunostaining of COLII in each group of the mouse model (n = 3). scale bars =50 um.

(G) Representative images of immunostaining of MMP13 in each group of the mouse model (n = 3), with yellow dashed line indicating the full thickness of the cartilage. scale bars =100 um.

(H) Representative images and quantification of staining of TUNEL in each group of the mouse model (n = 3), with yellow dashed line indicating the full thickness of the cartilage and yellow arrow indicating TUNEL-positive cells. scale bars =100 um.

Data are shown as the mean ± SD. Statistical significance was assessed using Kruskal-Wallis test (E, H) or one-way ANOVA (B). **P* < 0.05, ***P* < 0.01, ****P* < 0.001, *****P* < 0.0001.


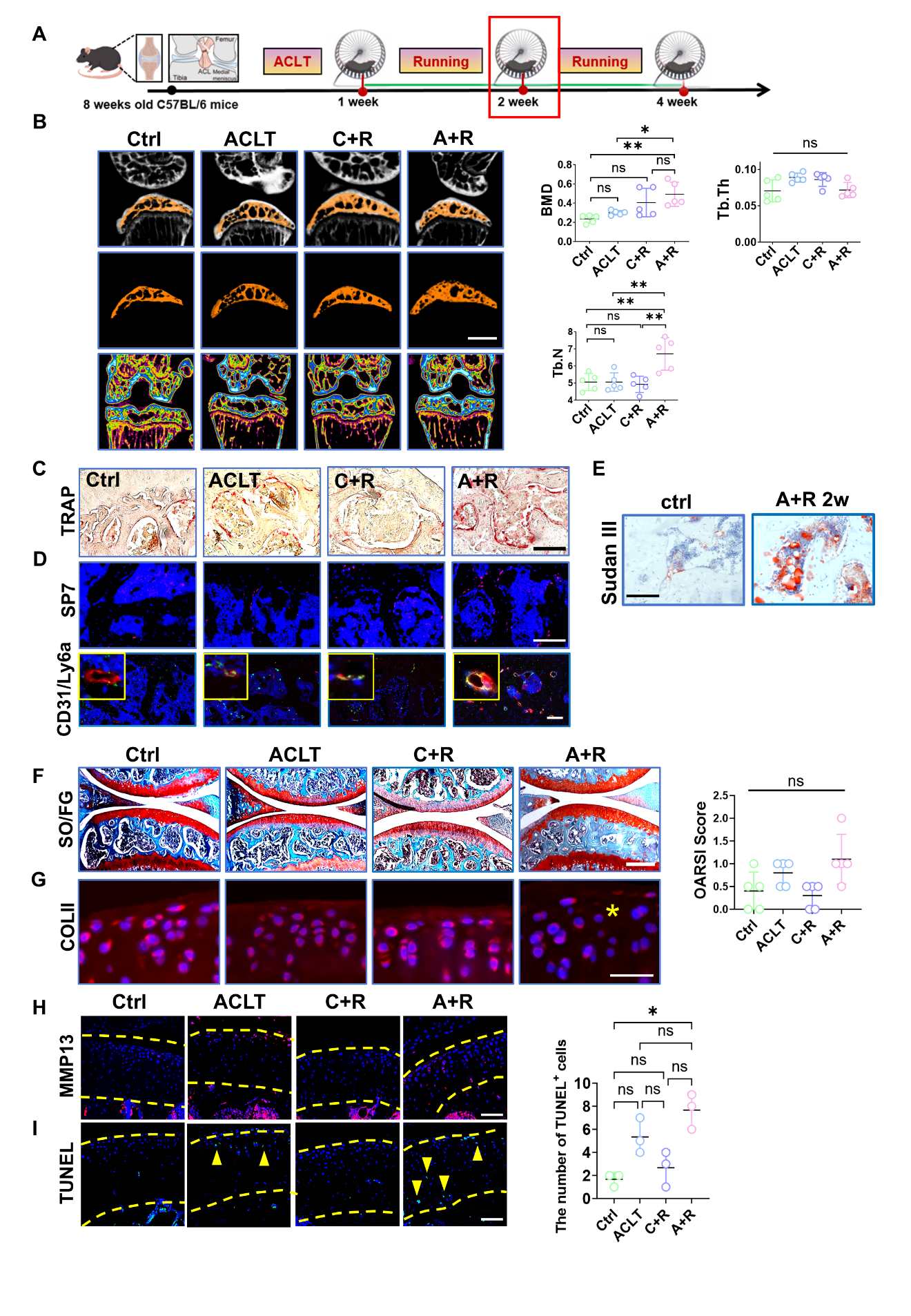


**Figure S2. Characterization of the BMLs animal model at 2 weeks post-modeling**

(A) Schematic diagram of constructing the combined anterior cruciate ligament transection (ACLT) and forced wheel running model (A+R) at two week post-modeling.

(B) Representative μCT images and quantification of the trabecular bone number (Tb. N.), trabecular bone thickness (Tb. Th.), and bone mineral density (BMD) in the subchondral bone of tibia from each group of the mouse model (n = 5). scale bars = 1 mm.

(C) Representative images of staining of tartrate resistant acid phosphatase (TRAP) in each group of the mouse model (n = 5). scale bars =50 um.

(D) Representative images of immunostaining of SP7 and CD31/Ly6a in each group of the mouse model (n = 3). scale bars =50 um.

(E) Representative images of staining of Sudan III in control and BML groups. scale bars =25 um.

(F) Representative images of Safranin-O in each group of the mouse model and quantification of OARSI scores (n = 5). scale bars =200 um.

(G) Representative images of immunostaining of COLII in each group of the mouse model (n = 3). * indicates degradation of the cartilage matrix. scale bars =50 um.

(H) Representative images of immunostaining of MMP13 in each group of the mouse model (n = 3), with yellow dashed line indicating the full thickness of the cartilage. scale bars =100 um.

(I) Representative images and quantification of staining of TUNEL in each group of the mouse model (n = 3), with yellow dashed line indicating the full thickness of the cartilage and yellow arrow indicating TUNEL-positive cells. scale bars =100 um.

Data are shown as the mean ± SD. Statistical significance was assessed using Kruskal-Wallis test (F, I) or one-way ANOVA (B). **P* < 0.05, ***P* < 0.01, ****P* < 0.001, *****P* < 0.0001.


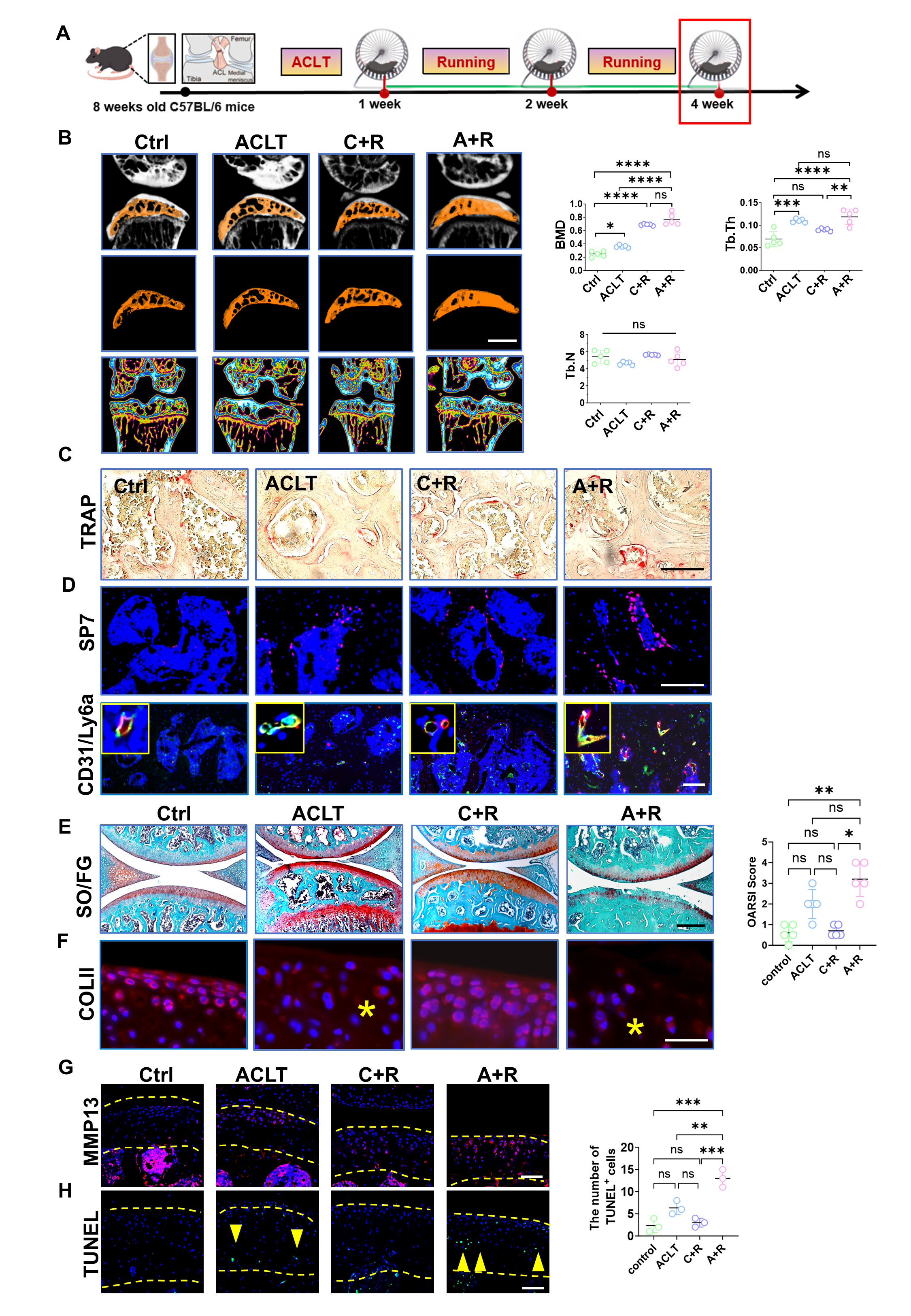


**Figure S3. Characterization of the BMLs animal model at 4 weeks post-modeling**

(A) Schematic diagram of constructing the combined anterior cruciate ligament transection (ACLT) and forced wheel running model (A+R) at four week post-modeling.

(B) Representative μCT images and quantification of the trabecular bone number (Tb. N.), trabecular bone thickness (Tb. Th.), and Bone Mineral Density (BMD) in the subchondral bone of tibia from each group of the mouse model (n = 5). scale bars = 1 mm.

(C) Representative images of staining of tartrate resistant acid phosphatase (TRAP) in each group of the mouse model (n = 5). scale bars =50 um.

(D) Representative images of immunostaining of SP7 and CD31/Ly6a in each group of the mouse model (n = 3). scale bars =50 um.

(E) Representative images of Safranin-O in each group of the mouse model and quantification of OARSI scores (n = 5). scale bars =200 um.

(F) Representative images of immunostaining of COLII in each group of the mouse model (n = 3). * indicates degradation of the cartilage matrix. scale bars =50 um.

(G) Representative images of immunostaining of MMP13 in each group of the mouse model (n = 3), with yellow dashed line indicating the full thickness of the cartilage. scale bars =100 um.

(H) Representative images and quantification of staining of TUNEL in each group of the mouse model (n = 3), with yellow dashed line indicating the full thickness of the cartilage and yellow arrow indicating TUNEL-positive cells. scale bars =100 um.

Data are shown as the mean ± SD. Statistical significance was assessed using Kruskal-Wallis test (E) or one-way ANOVA (B, H). **P* < 0.05, ***P* < 0.01, ****P* < 0.001, *****P* < 0.0001.

**
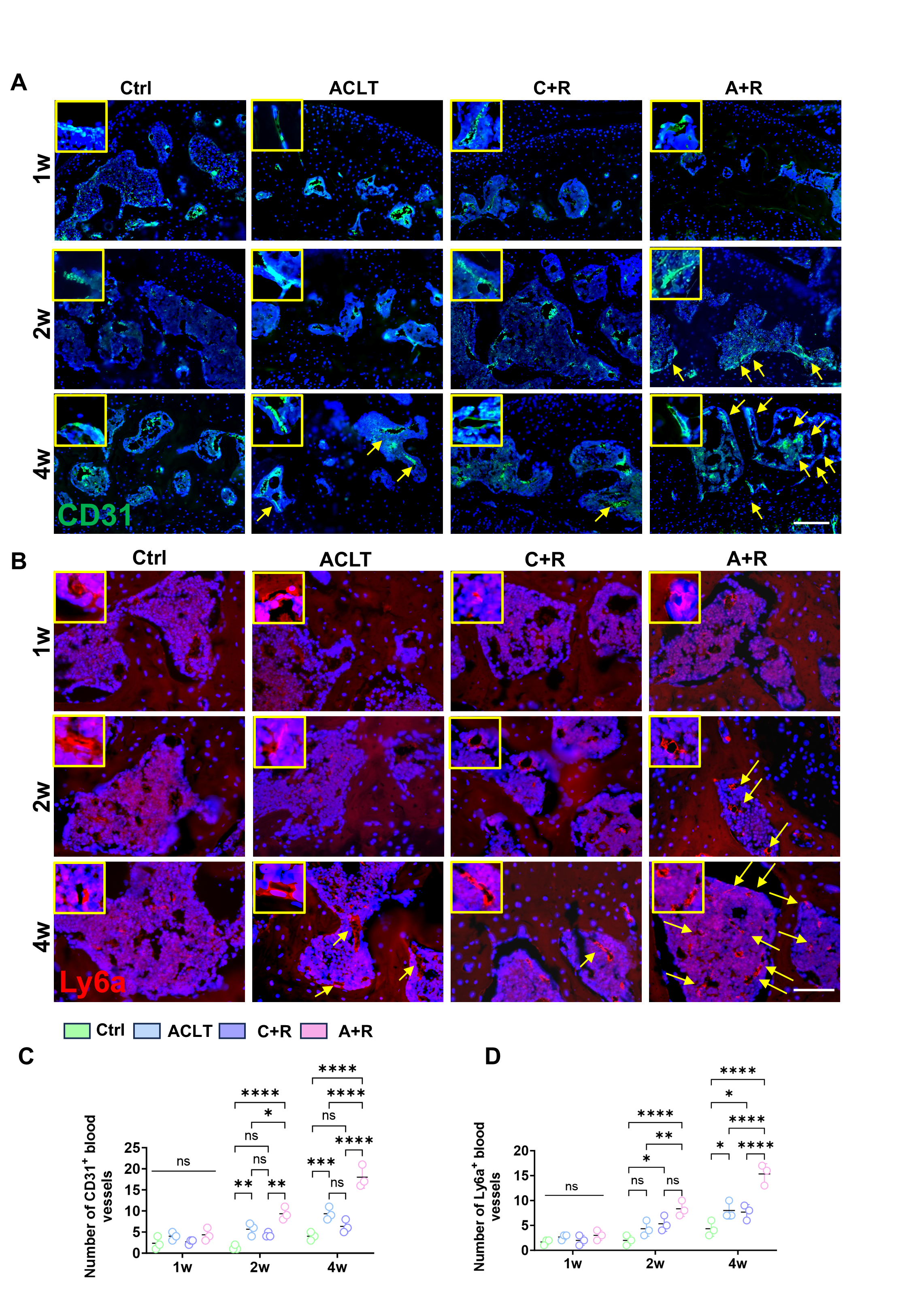
Figure S4. Characterization of S-type vessels in subchondral bone**

(A and C) Representative images and quantification of immunostaining of CD31 in each group of subchondral bone (n = 3). scale bars =100 um.

(B and D) Representative images and quantification of immunostaining of Ly6a in each group of subchondral bone (n = 3). scale bars =50 um.

Data are shown as the mean ± SD. Multiple group comparison were assessed using Two-way ANOVA. **P* < 0.05, ***P* < 0.01, ****P* < 0.001, *****P* < 0.0001.


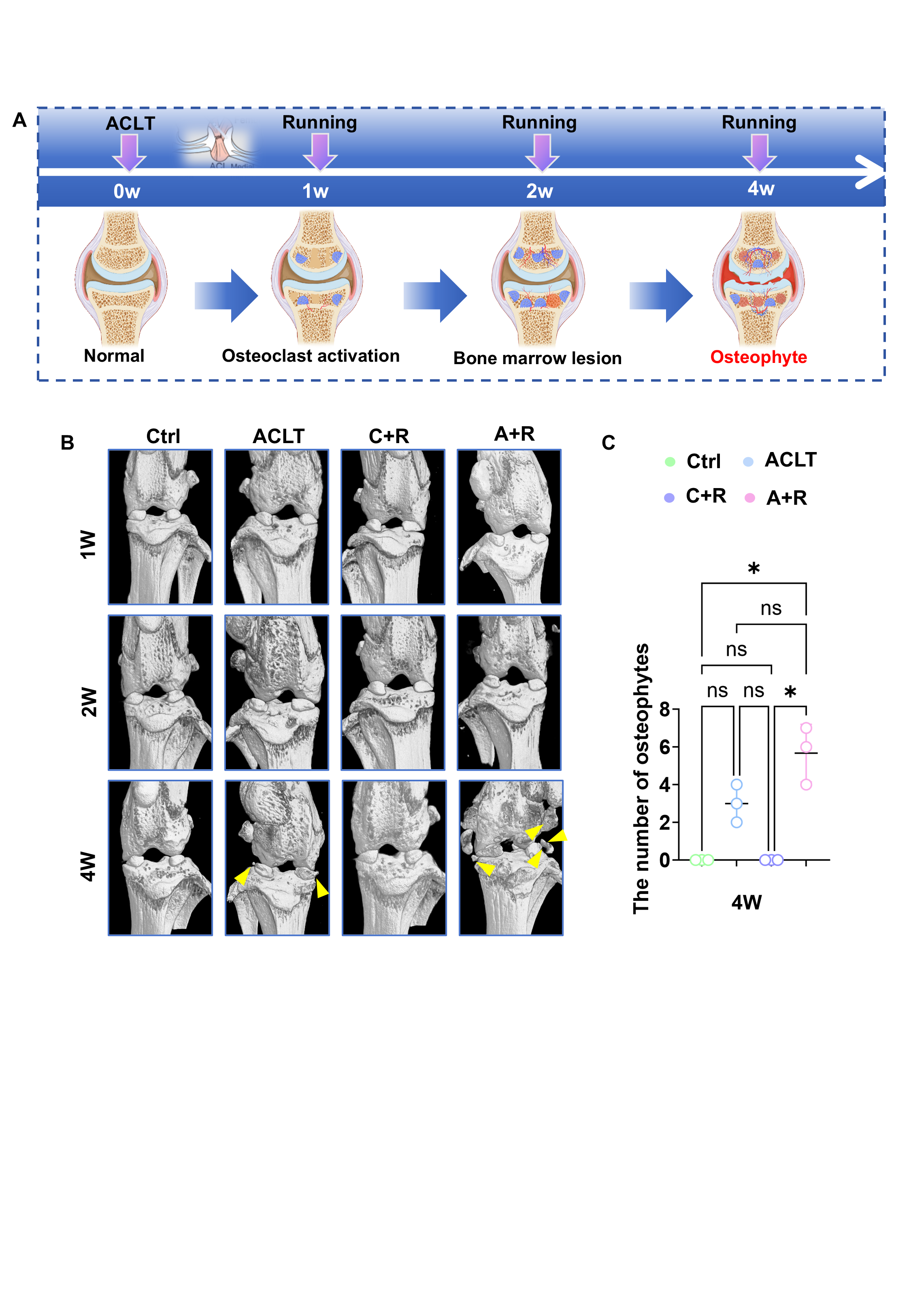


**Figure S5. Representative 3D reconstructed μ-CT images**

(A) Schematic illustration of pathological development of mice after modeling.

(B) Representative 3D reconstructed μCT images of the knee joints from each group of the mouse model, with yellow arrow indicating osteophytes.

(C) Quantification of the number of osteophytes at 4-week in (B) (n = 5).

Data are shown as the mean ± SD. Multiple group comparison were assessed using Kruskal-Wallis test. **P* < 0.05, ***P* < 0.01, ****P* < 0.001, *****P* < 0.0001.


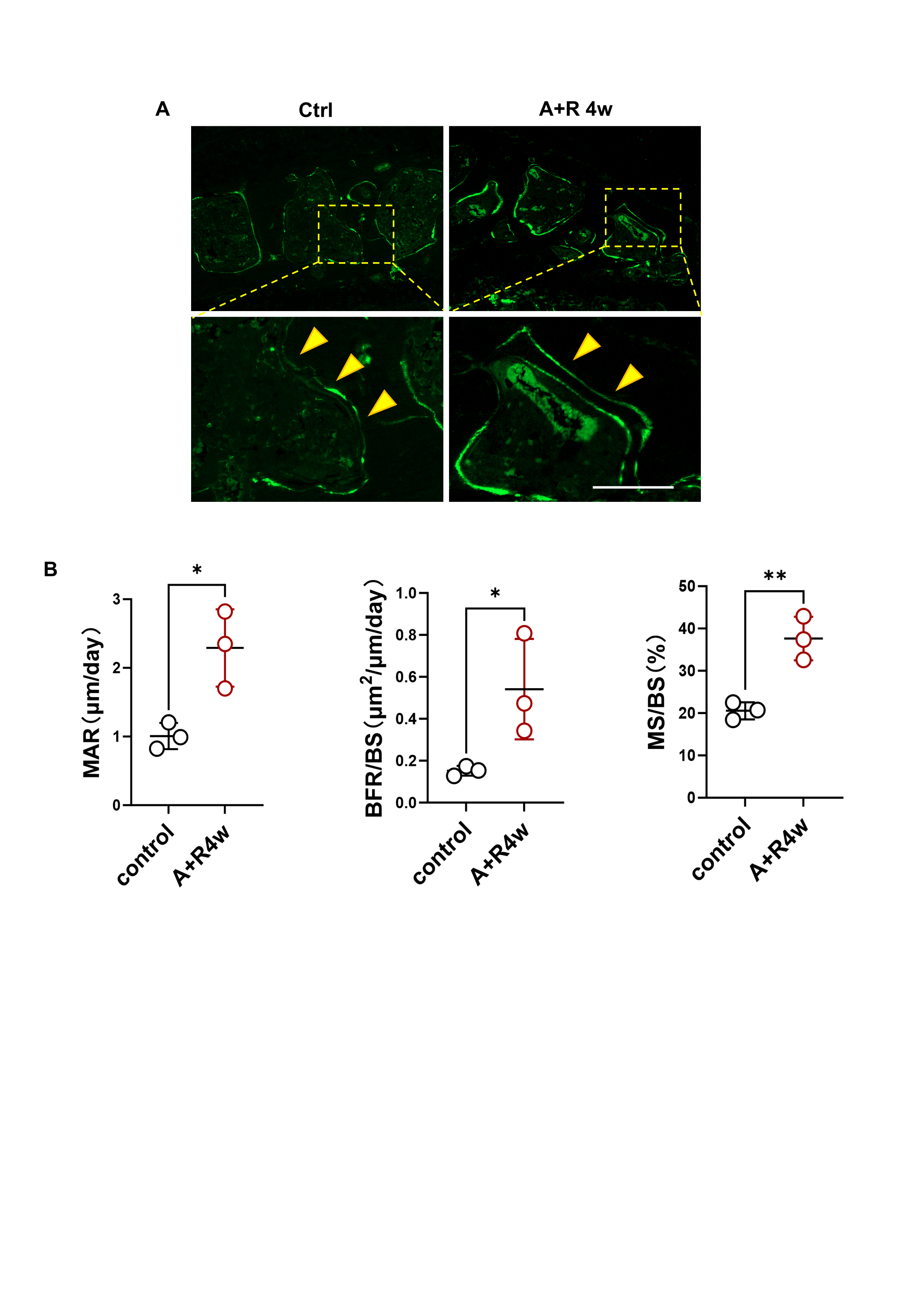


**Figure S6.** Representative images (A) and quantification (B) of calcein double labeling parameters (n = 3). scale bars =50 um. Data are shown as the mean ± SD. Statistical significance was assessed using Student’s t test. **P* < 0.05, ***P* < 0.01, ****P* < 0.001, *****P* < 0.0001.


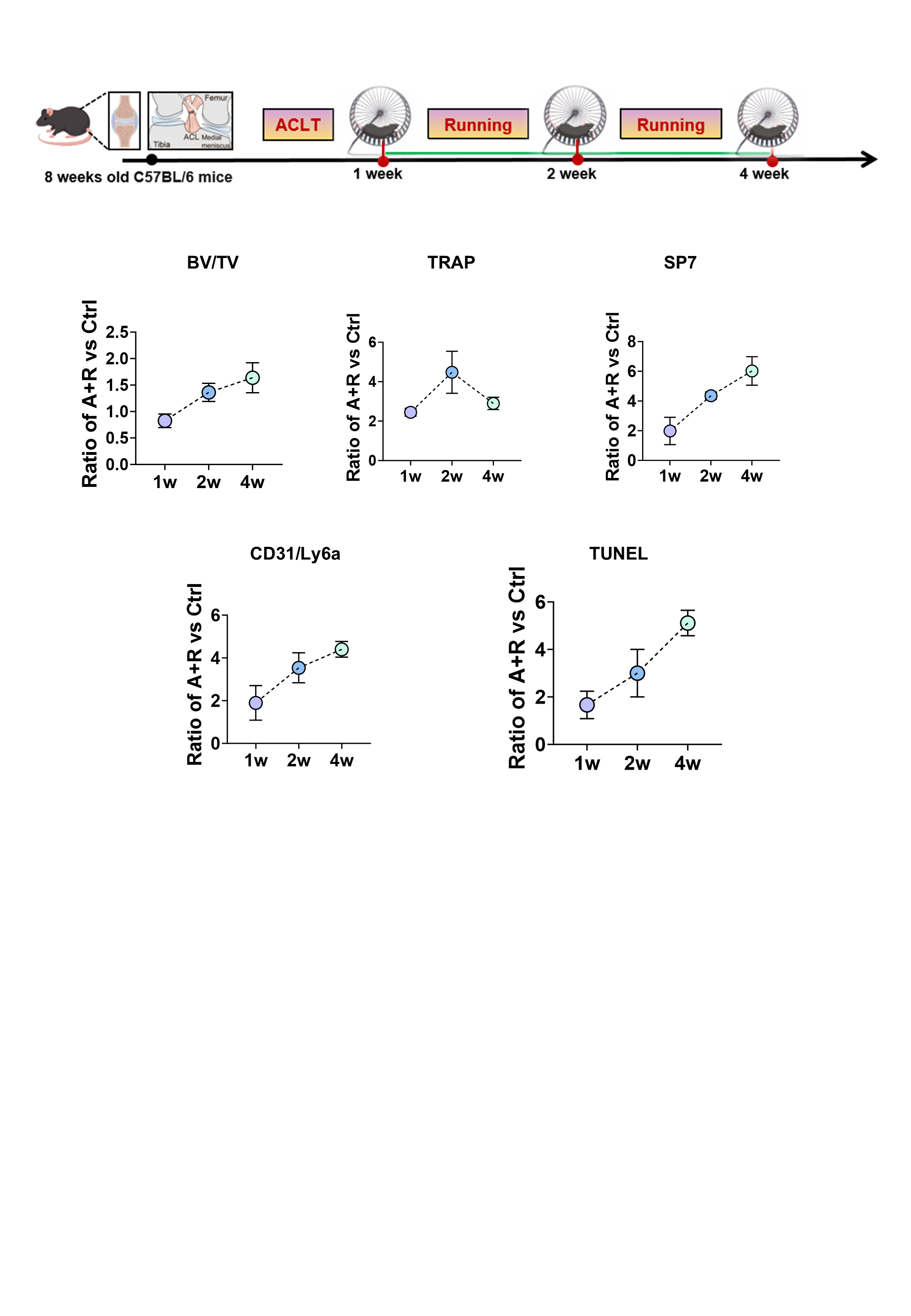


**Figure S7. Normalized parameters of A+R group to controls**

After 1-week, 2-weeks, and 4-weeks of modeling, dynamic curves were plotted for each indicator, including bone volume/trabecular bone volume ratio (BV/TV), tartrate resistant acid phosphatase (TRAP), SP7, CD31/Ly6a, and TUNEL (n = 5 for BV/TV and TRAP; n = 3 for SP7, CD31/Ly6a, and TUNEL).


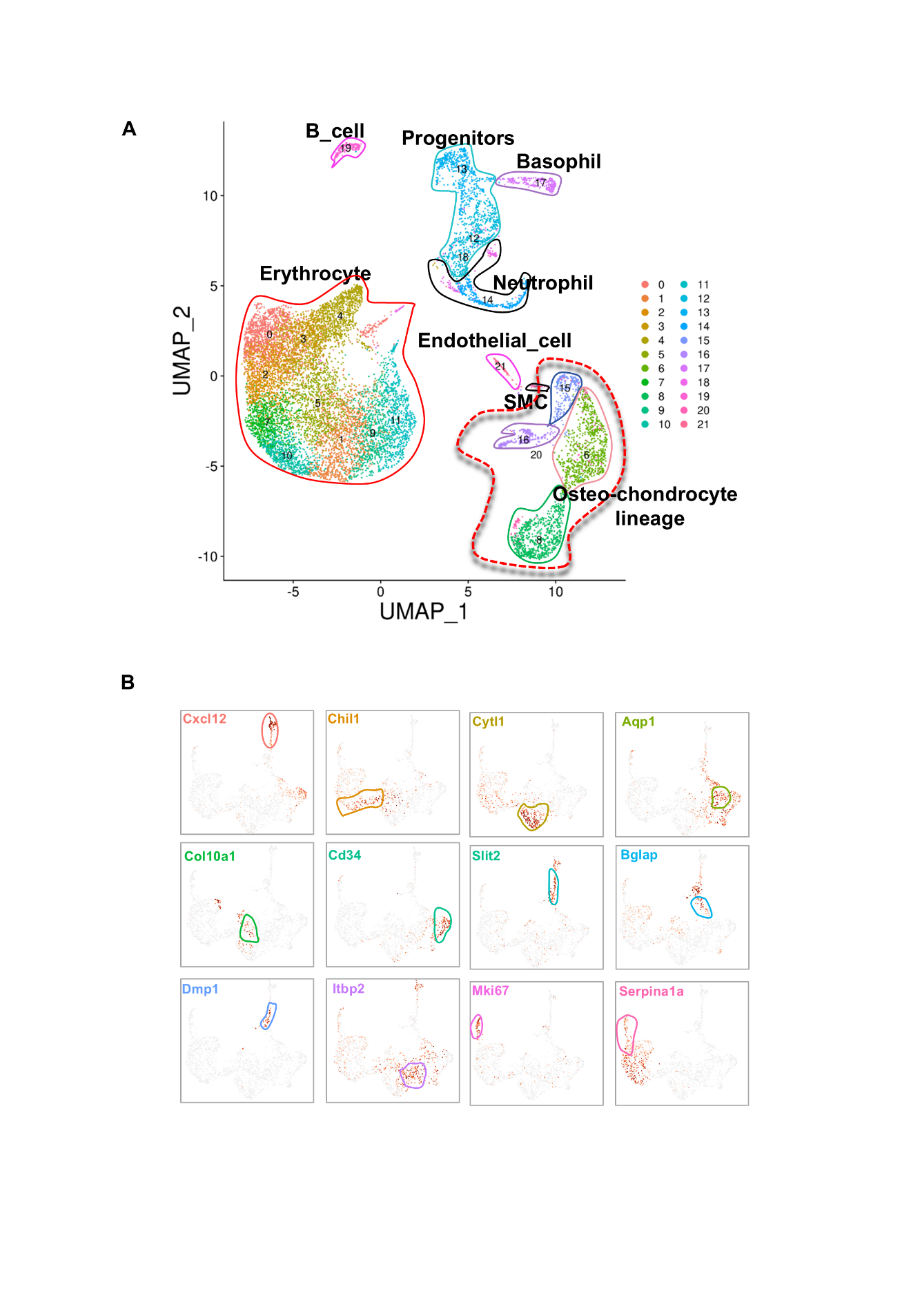


**Figure S8. Visualisation of UMAP plot coloured according to cell types for mice OA osteochondral single-cell transcriptomes**

(A) Single-cell transcriptome profiles of mice osteochondral tissue during BML development (no cells were removed).

(B) Dot plots showing the expression of indicated markers for each cell cluster on the UMAP plot.


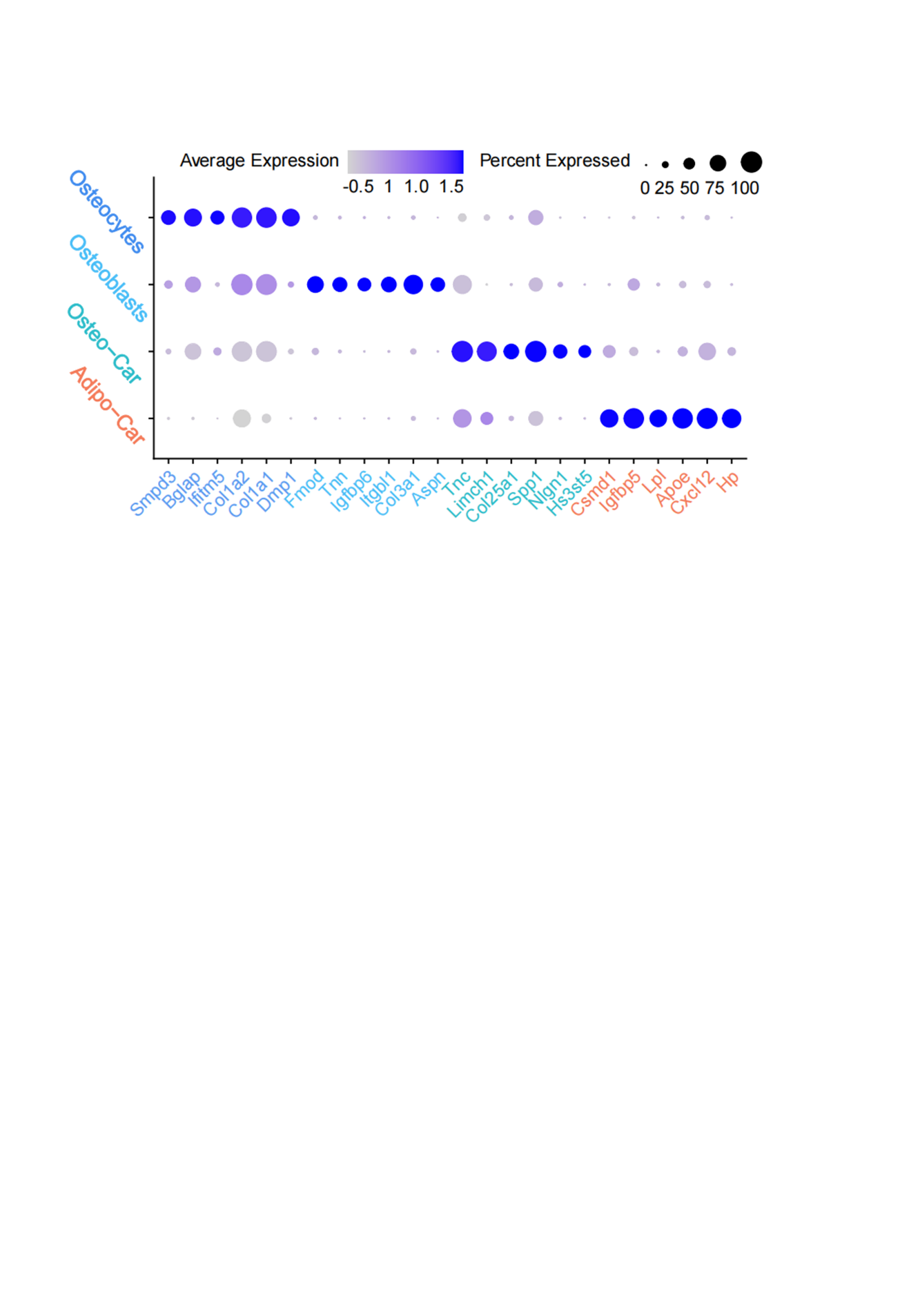


**Figure S9.** The top six representative markers of the bone cells subpopulation.

**
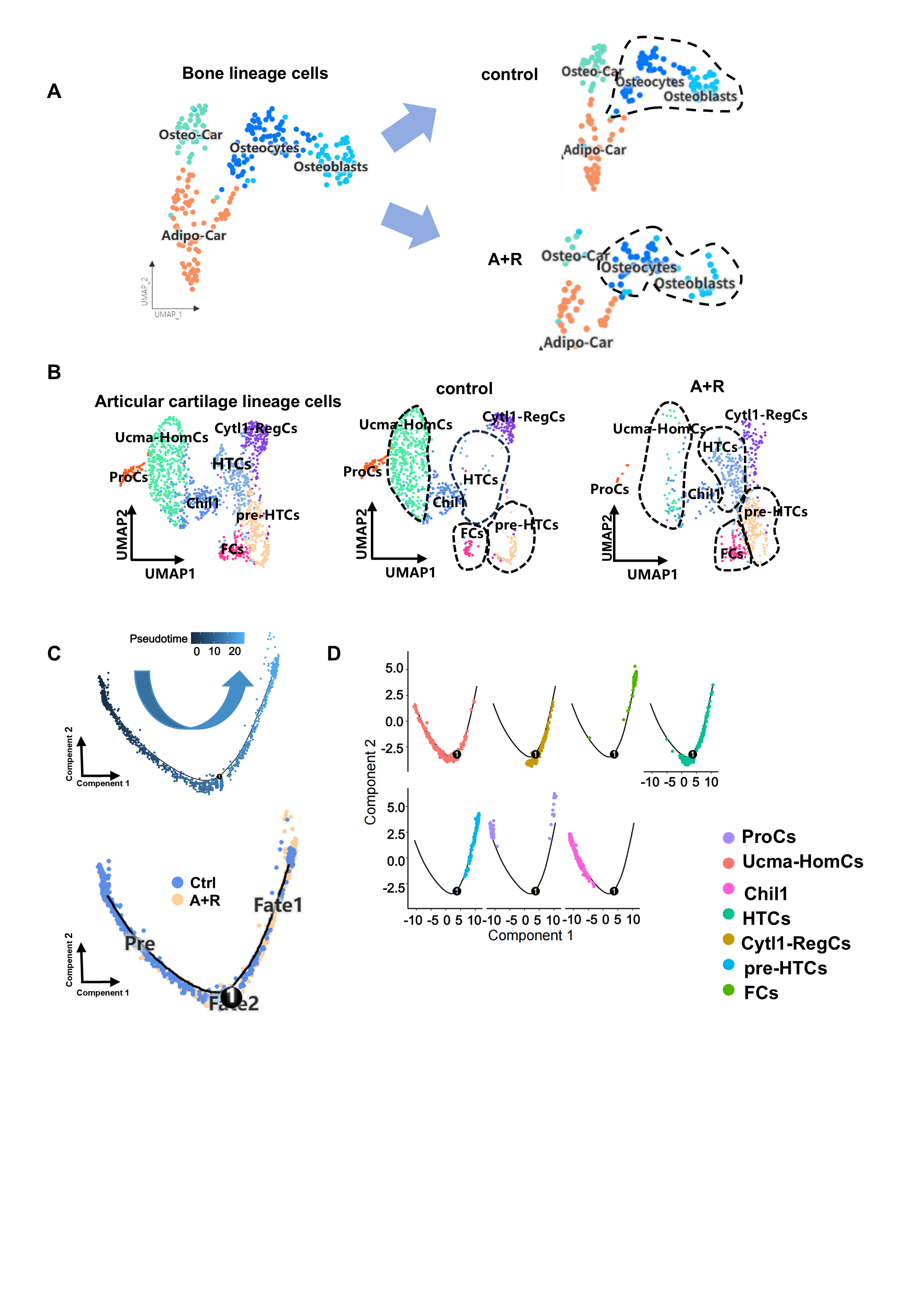
**

**Figure S10. UMAP plots and pseudo-time analysis of bone cell and chondrocyte lineage** **in single-cell RNA sequencing**

(A) UMAP plot of bone cell lineage cells from BML and control samples.

(B) UMAP plot of **chondrocyte** lineage cells from BML and control samples.

(C-D) Pseudotemporal annotation of the **chondrocyte** developmental tree.


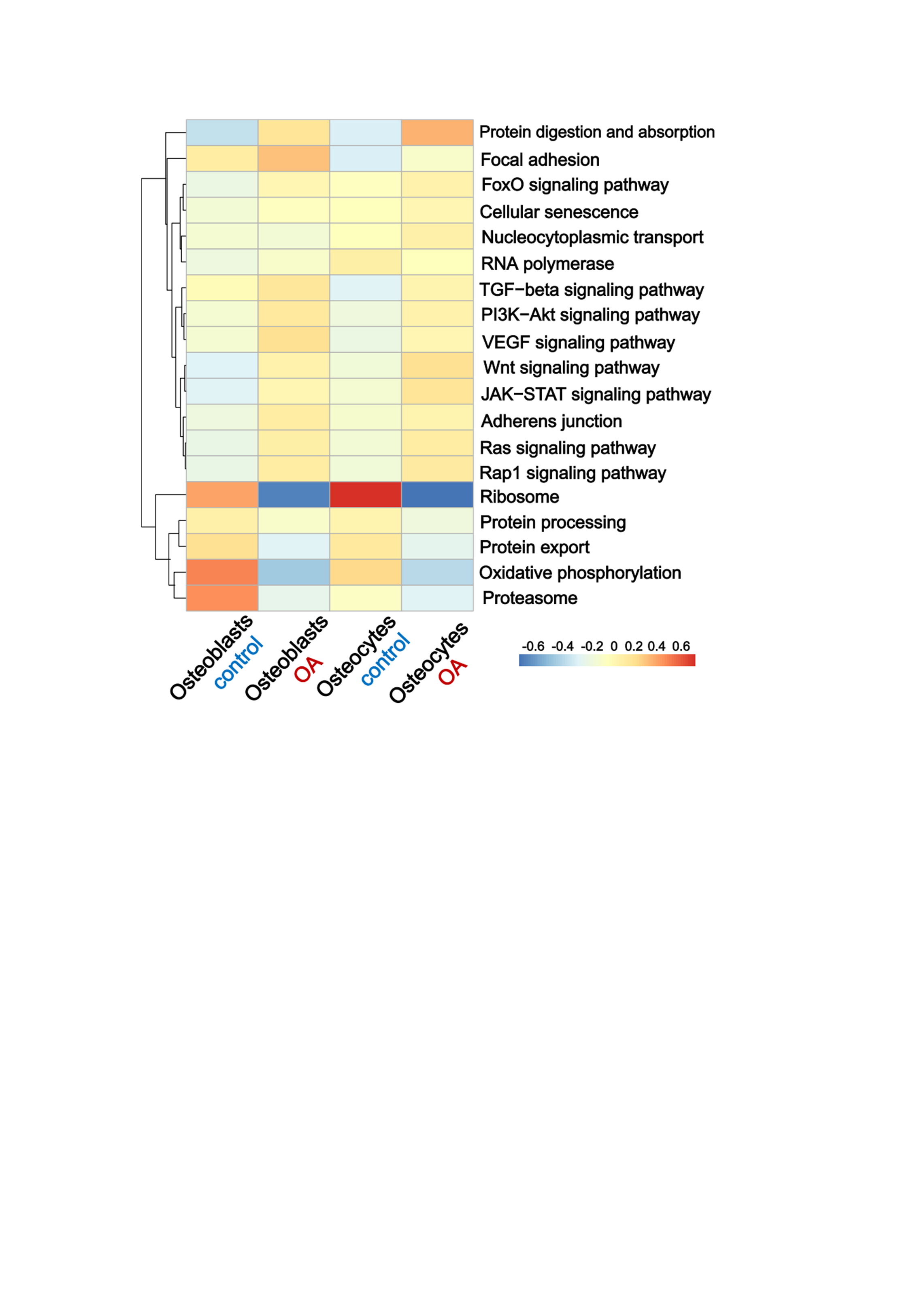


**Figure S11.** Kyoto Encyclopedia of Genes and Genomes **(**KEGG) pathway enrichment analysis identifies differentially regulated signaling pathways in BML versus control groups.


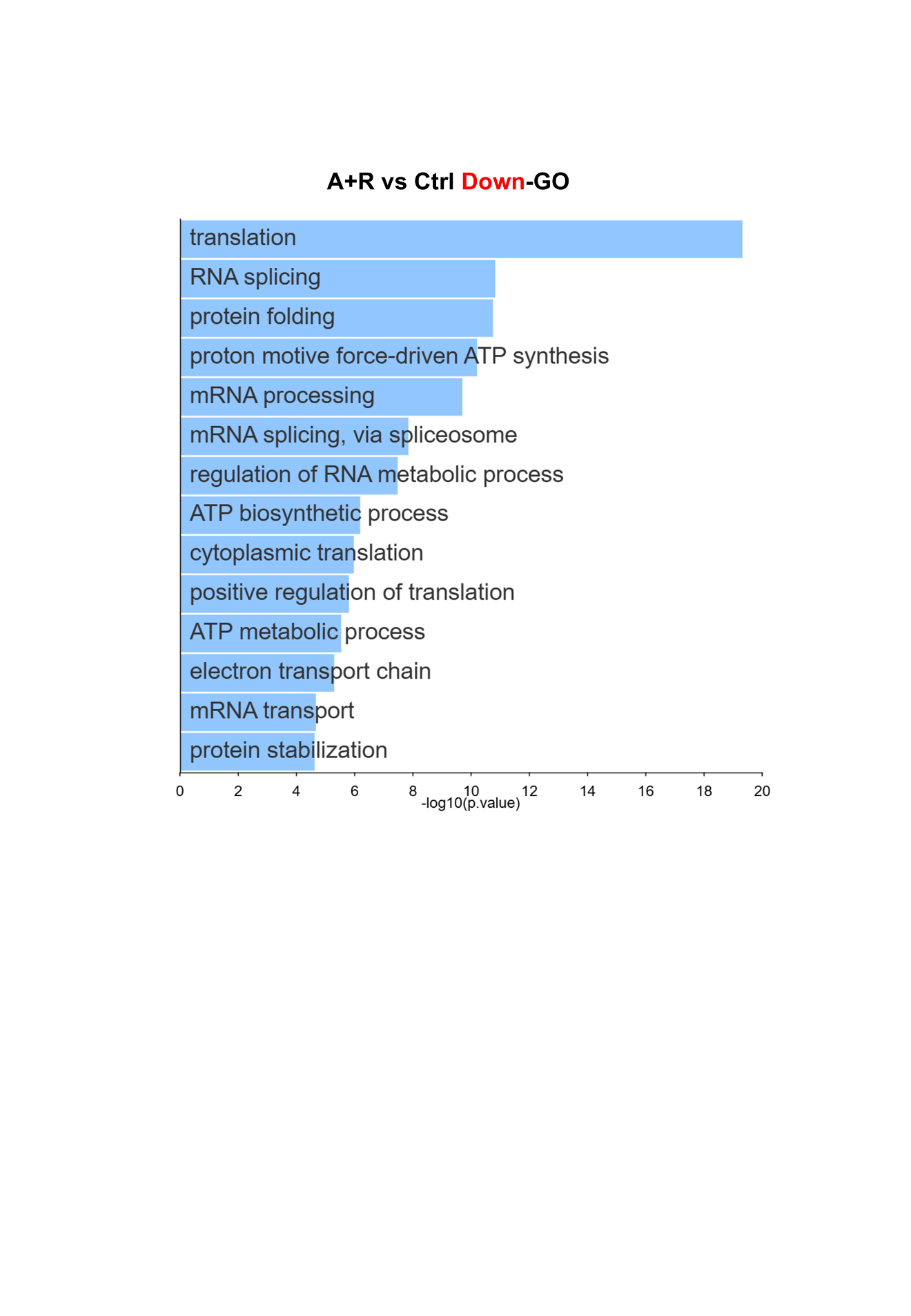


**Figure S12.** The significant Gene Ontology (GO) terms were enriched by the downregulated Differential expressed genes (DEGs) in chondrocytes from BML groups compared to control groups.


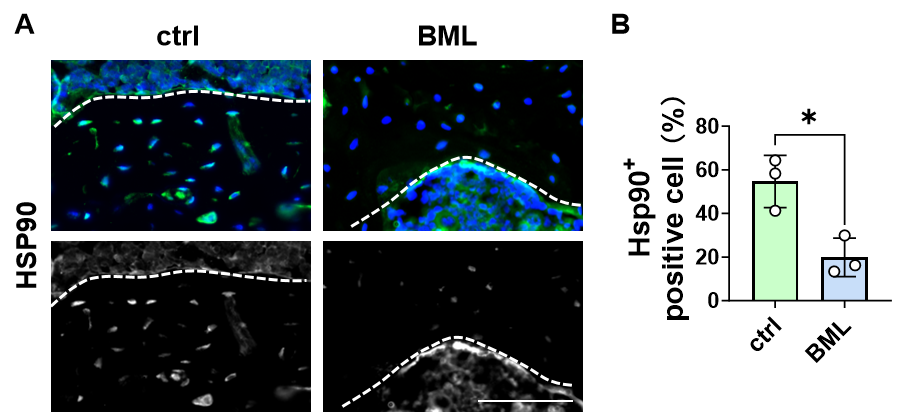


**Figure S13.** Representative images (A) and quantification (B) of immunostaining of HSP90 at the 4-week in control, and BML groups (n = 3). The white dashed lines mark the junction of the marrow cavity with the trabecular bone. scale bars =25 um. Data are shown as the mean ± SD. Statistical significance was assessed using Student’s t test. **P* < 0.05, ***P* < 0.01, ****P* < 0.001, *****P* < 0.0001.


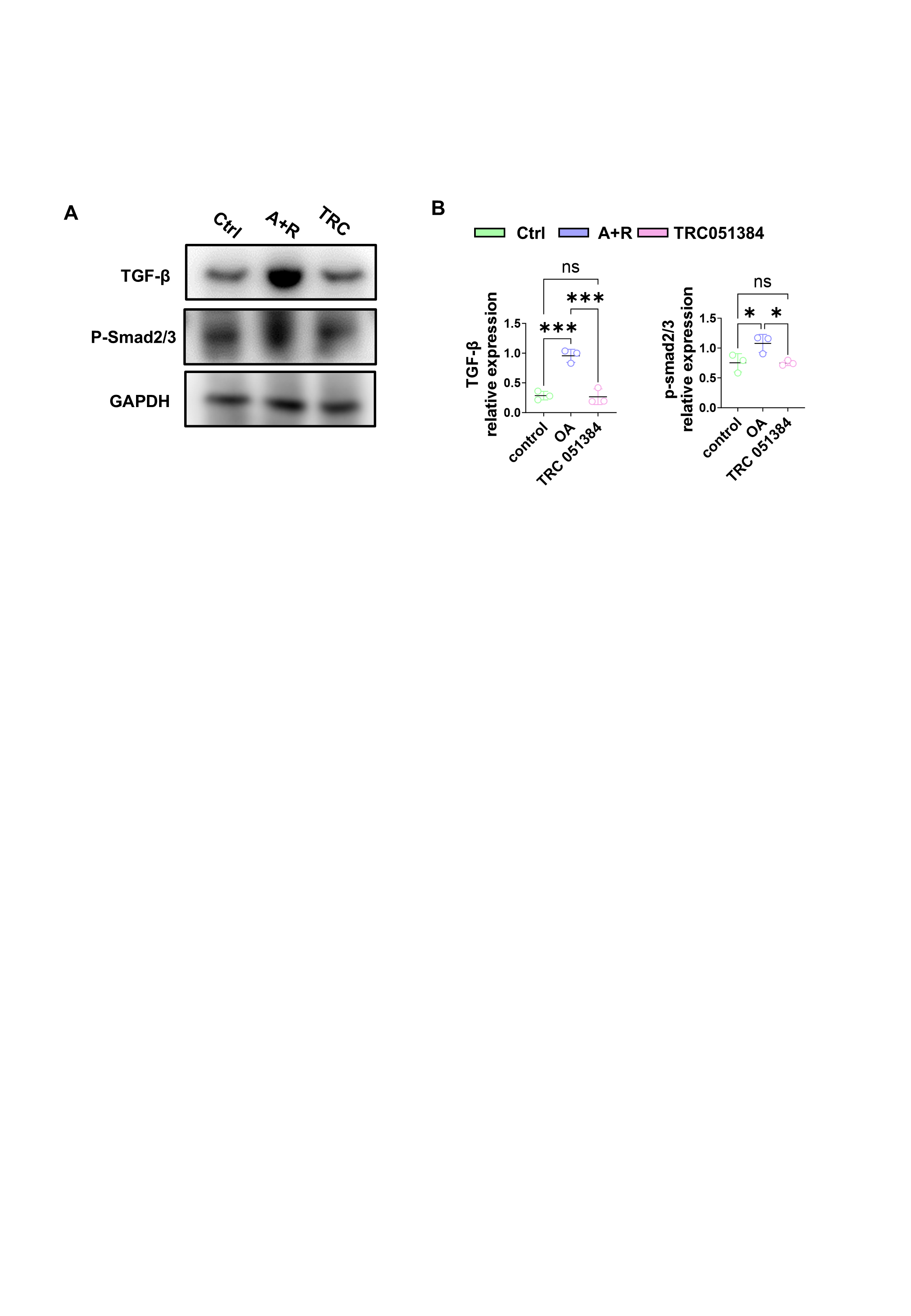


**Figure S14. Altered TGF-β pathway in control, BML, and treatment groups**

1. Western blot analysis of TGF-β and P-Smad2/3 expression in control, BML, and treatment groups.
2. Quantitative statistical analysis of Western blot results in (A) (n = 3).

Data are shown as the mean ± SD. Statistical significance was assessed using one-way ANOVA. **P* < 0.05, ***P* < 0.01, ****P* < 0.001, *****P* < 0.0001.


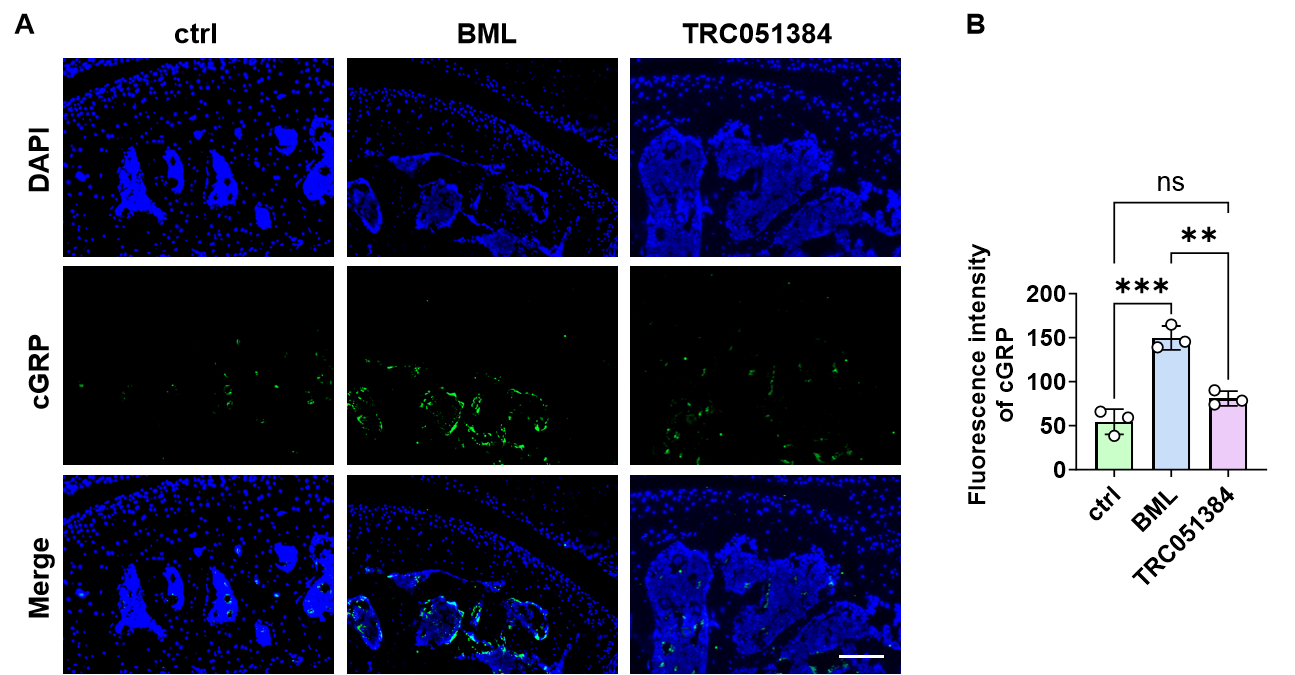


**Figure S15.** Representative images (A) and quantification (B) of immunostaining of cGRP at the 4-week in control, BML, and treatment groups (n = 3). scale bars =100 um. Data are shown as the mean ± SD. Statistical significance was assessed using one-way ANOVA. **P* < 0.05, ***P* < 0.01, ****P* < 0.001, *****P* < 0.0001.


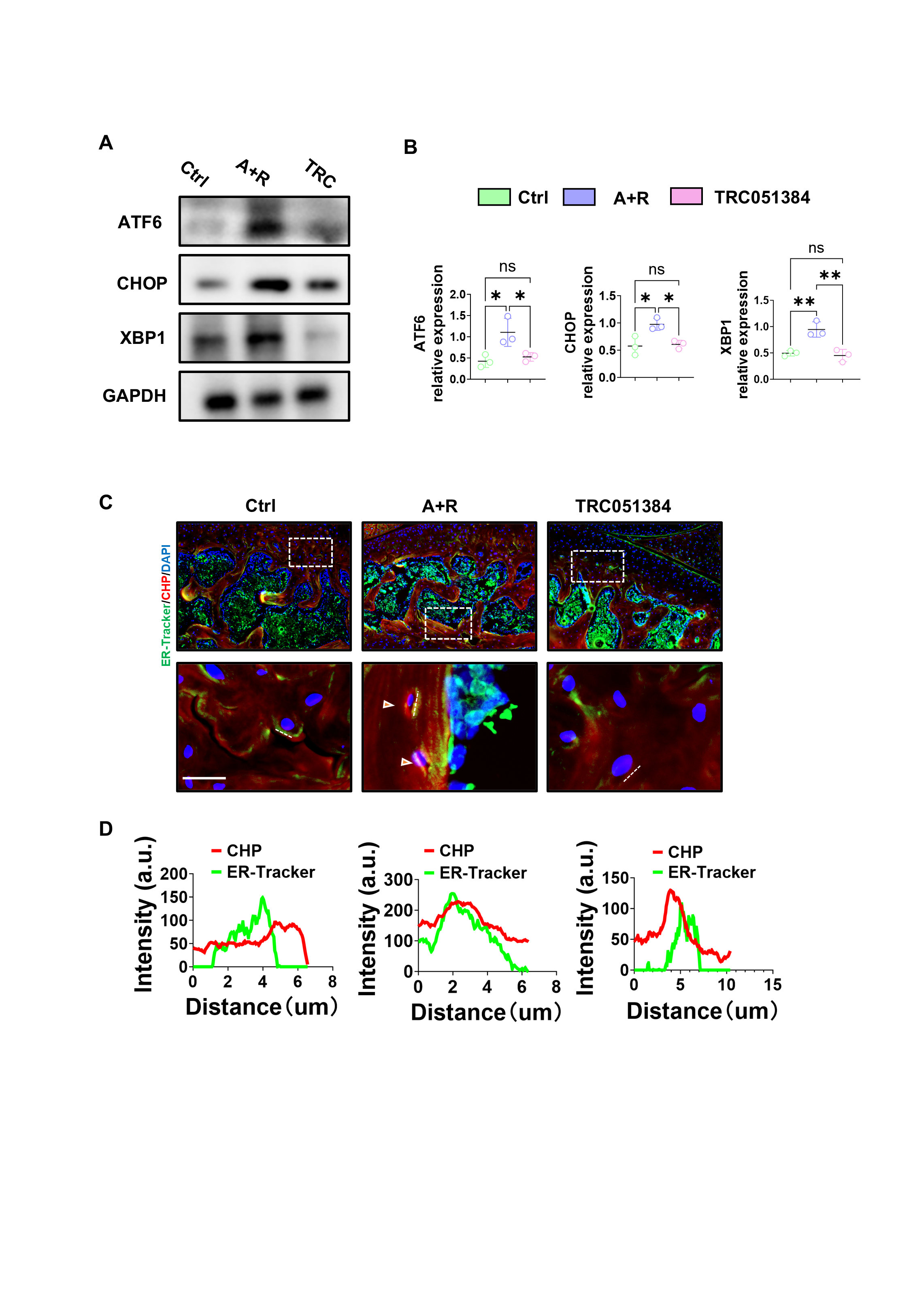


**Figure S16. Misfolded proteins trigger endoplasmic reticulum stress**

(A-B) Western blot analysis (A) and quantification (B) of ATF6, CHOP, and XBP1 in subchondral bone tissue proteins from control, BML, and treatment groups (n = 3).

(C) Representative images of co-localized fluorescent staining for **ER tracer (ER-Tracker™ Green)** and **Collagen hybridizing peptide (CHP)**, and the white dotted line around the nucleus indicates the location of the fluorescence intensity to be measured. (scale bars =50 um).

(D) Statistical analysis of fluorescence intensity of the distribution of ER-Tracker/CHP co-localization in the cells shown in (N) (n = 3).

Data are shown as the mean ± SD. Statistical significance was assessed using one-way ANOVA. **P* < 0.05, ***P* < 0.01, ****P* < 0.001, *****P* < 0.0001.


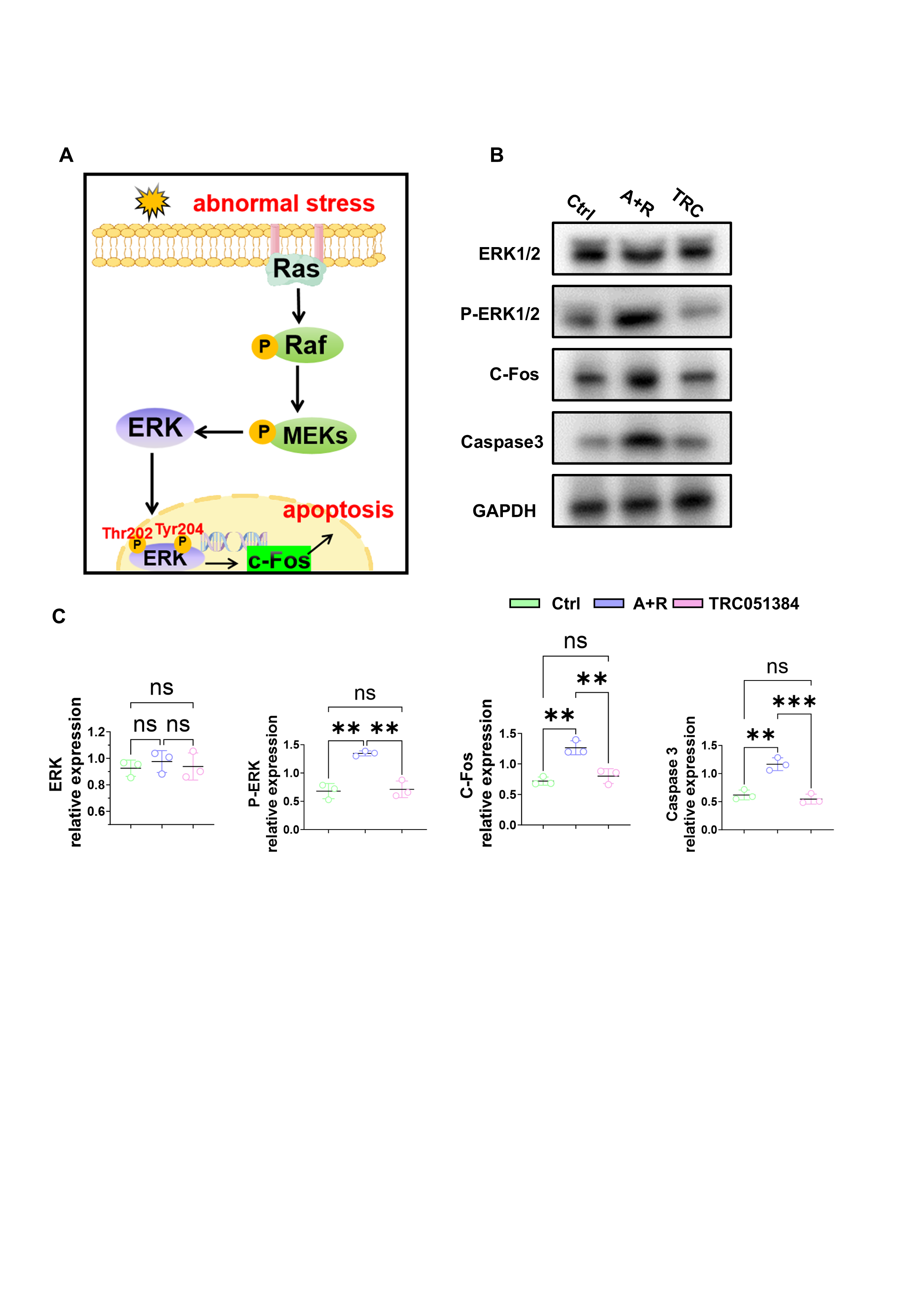


**Figure S17. Ras signaling pathway regulates the apoptosis of bone cells**

(A) Schematic illustration of the mechanism of Ras pathway leading to apoptosis in bone cells.

(B-C) Western blot analysis (B) and quantification (C) of ERK1/2, P-ERK1/2, C-Fos, and Caspase3 in subchondral bone tissue proteins from control, BML, and treatment groups (n = 3).

Data are shown as the mean ± SD. Statistical significance was assessed using one-way ANOVA. **P* < 0.05, ***P* < 0.01, ****P* < 0.001, *****P* < 0.0001.


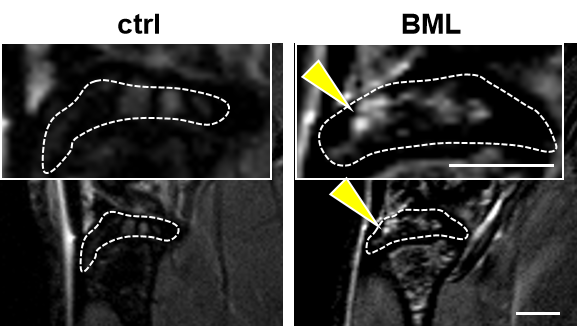


**Figure S18.** Representative images of 9.4T MRI sequences of T2 phase fat suppression in ctrl and BML group, with yellow arrows indicating BML region and white dotted line indicating subchondral bone region. The genotypes of mice in both the control and BML groups were *Col10a1-Cre; R26^tdt+^*. Scale bar = 1 mm.


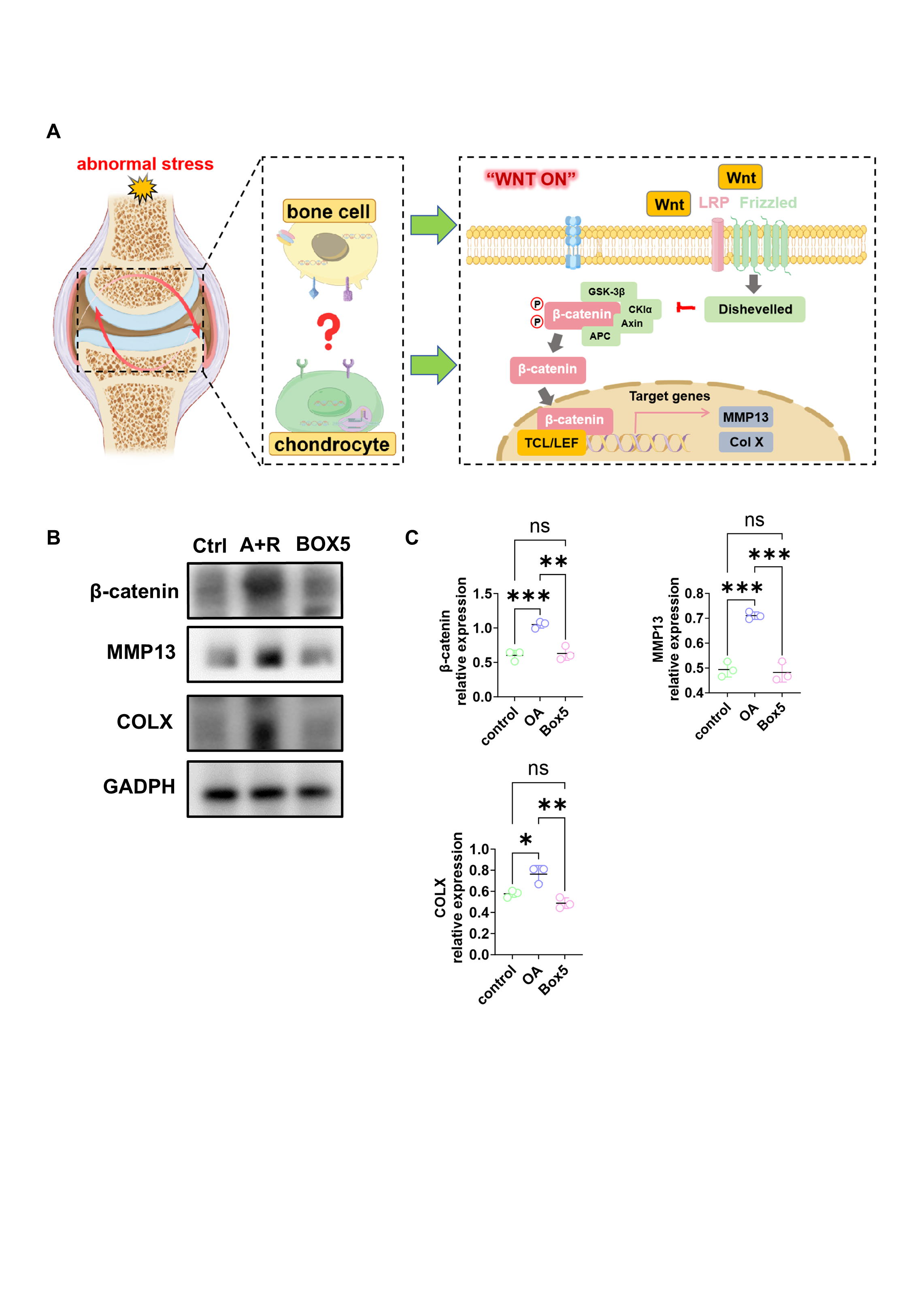


**Figure S19. Communication between bone cells and chondrocytes**

(A) Schematic diagram of subchondral bone and cartilage crosstalk.

(B-C) Western blot analysis (B) and quantification (C) of β-catenin, MMP13, COLX in cartilage tissue proteins in control, A+R and treatment samples (n = 3).

Data are shown as the mean ± SD. Statistical significance was assessed using one-way ANOVA. **P* < 0.05, ***P* < 0.01, ****P* < 0.001, *****P* < 0.0001.


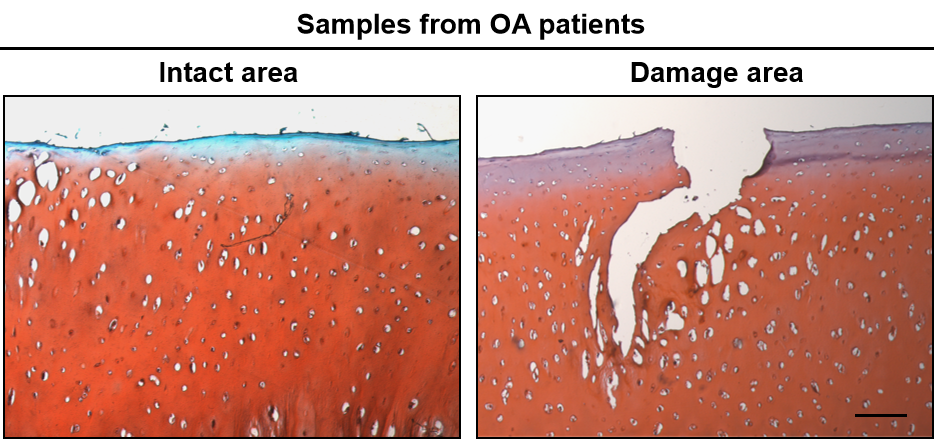


**Figure S20.** Representative histological images of intact and damaged regions in knee articular cartilage from OA patients. Scale bar = 100 μm.

**Supplementary Table 1. Detailed information for antibodies or drugs**

| **Antibody or drugs** | **Brand** | **Catalog No.** | **concentration** | **Application** |
| --- | --- | --- | --- | --- |
| **anti-TRAP** | [Solarbio](https://www.solarbio.com/goodsInfo?id=18432) | G1492 | 0.5mM | IHC |
| **anti-SP7** | Abcam | ab209484 | 1:200 | IF |
| **anti-CD31** | R&D | AF3628 | 1:100 | IF |
| **anti-LY6A** | BDPharmingen™ | 553333 | 1:100 | IF |
| **anti-MMP13** | Proteintech | 18165-1-AP | 1:200 | IF |
| **anti-COLII** | Abcam | ab34712 | 1:200 | IF |
| **anti-HSP70** | Proteintech | 10995-1-AP | 1:100 | IF |
| **anti-PSMA6** | Proteintech | 11573-1-AP | 1:50 | IF/WB |
| **anti-PSMB1** | Proteintech | 11749-1-AP | 1:50 | IF/WB |
| **anti-PSMA5** | Proteintech | 19178-1-AP | 1:50 | IF/WB |
| **anti-PSMB8** | Proteintech | 14859-1-AP | 1:50 | IF |
| **anti-COLI** | Affinity | AF7001 | 1:200 | IF/WB |
| **anti-USP19** | Proteintech | 25768-1-AP | 1:100 | IF/WB |
| **anti-WNT5A** | Proteintech | 55184-1-AP | 1:100 | IF |
| **anti-β-catenin** | Proteintech | 51067-2-AP | 1:100 | IF/WB |
| **anti-COLX** | Proteintech | 26984-1-AP | 1:200 | IF/WB |
| **anti-TGF-β** | Proteintech | 81746-2-RR | 1:200 | WB |
| **anti-P-Smad2/3** | Proteintech | 80427-2-RR | 1:50 | WB |
| **anti-ATF6** | ABclonal | A0202 | 1:200 | WB |
| **anti-CHOP** | ABclonal | A20987 | 1:200 | WB |
| **anti-XBP1** | ABclonal | A25319 | 1:200 | WB |
| **anti-ERK1/2** | ABclonal | A4782 | 1:200 | WB |
| **anti-P-ERK1/2** | ABclonal | AP0234 | 1:200 | WB |
| **anti-c-FOS** | Proteintech | 66590-1-lg | 1:200 | WB |
| **anti-Caspase3** | Proteintech | 25128-1-AP | 1:200 | WB |
| **TRC051384** | MedChemExpress | [867164-40-7](https://www.medchemexpress.cn/cas/867164-40-7.html) | 5mg/ml | Treatment |
| **BOX5** | MedChemExpress | [1206604-29-6](https://www.medchemexpress.cn/cas/1206604-29-6.html) | 5mg/ml | Treatment |
| **EDTA buffer** | [Solarbio](https://www.solarbio.com/goodsInfo?id=18432) | E1171 | 10% | Decalcification |

**Supplementary Table 2. Clinical and demographic characteristics of the patients**

| **Patient ID** | **Age (years)** | **Gender** | **Sampled tibial plateau** | **Kellgren-Lawrence (KL)** | **International Cartilage Repair Society（ICRS）** | | **Diagnosis** |
| --- | --- | --- | --- | --- | --- | --- | --- |
|  |  |  |  |  | **Damage area** | **Intact area** |  |
| Case 1 | 66 | Male | Right | III | III | I | Knee OA |
| Case 2 | 64 | Male | Right | II | II | I | Knee OA |
| Case 3 | 58 | Female | Right | II | II | I | Knee OA |
| Case 4 | 74 | Female | Left | III | III | I | Knee OA |
| Case 5 | 69 | Female | Right | III | III | I | Knee OA |

**Supplementary Table 3. Sequences of primers**

| ***Usp19*-shRNA1** | **Forward** | GATCCCGATCCTTTGAAGCTGAGATTCTCGAGAATCTCAGCTTCAAAGGATCGTTTTTG |
| --- | --- | --- |
|  | **Recerse** | AGCTCAAAAACGATCCTTTGAAGCTGAGATTCTCGAGAATCTCAGCTTCAAAGGATCGG |
| ***Usp19*-shRNA2** | **Forward** | GATCCCGGCACAAGATGAGAAATGATCTCGAGATCATTTCTCATCTTGTGCCGTTTTTG |
|  | **Recerse** | AGCTCAAAAACGGCACAAGATGAGAAATGATCTCGAGATCATTTCTCATCTTGTGCCGG |
| ***Usp19*-shRNA3** | **Forward** | GATCCCGGTGACAACAGTAGACGAAACTCGAGTTTCGTCTACTGTTGTCACCGTTTTTG |
|  | **Recerse** | AGCTCAAAAACGGTGACAACAGTAGACGAAACTCGAGTTTCGTCTACTGTTGTCACCGG |

**Reference**

[1] S. Guo, Y. Huang, Y. Zhang, et al., “Impacts of exercise interventions on different diseases and organ functions in mice”, *Journal of Sport and Health Science*, **2020**, *9* (1), 53-73, <https://doi.org/10.1016/j.jshs.2019.07.004>.

[2] R. U. Kleemann, D. Krocker, A. Cedraro, J. Tuischer, G. N. Duda, “Altered cartilage mechanics and histology in knee osteoarthritis: relation to clinical assessment (ICRS Grade)”, *Osteoarthritis and Cartilage*, **2005**, *13* (11), 958-963, <https://doi.org/10.1016/j.joca.2005.06.008>.

[3] K. P. H. Pritzker, S. Gay, S. A. Jimenez, et al., “Osteoarthritis cartilage histopathology: grading and staging”, *Osteoarthritis and Cartilage*, **2006**, *14* (1), 13-29, <https://doi.org/10.1016/j.joca.2005.07.014>.

[4] U. Mendibil, R. Ruiz-Hernandez, S. Retegi-Carrion, et al., “Tissue-Specific Decellularization Methods: Rationale and Strategies to Achieve Regenerative Compounds”, *International Journal of Molecular Sciences*, **2020**, *21* (15), 5447, https://doi.org/10.3390/ijms21155447.

[5] D. W. Dempster, J. E. Compston, M. K. Drezner, et al., “Standardized nomenclature, symbols, and units for bone histomorphometry: a 2012 update of the report of the ASBMR Histomorphometry Nomenclature Committee”, *Journal of Bone and Mineral Research*, **2013**, *28* (1), 2-17, <https://doi.org/10.1002/jbmr.1805>.

[6] K. Hatzikotoulas, L. Southam, L. Stefansdottir, et al., “Translational genomics of osteoarthritis in 1,962,069 individuals”, *Nature*, **2025**, *641* (8065), 1217-1224, <https://doi.org/10.1038/s41586-025-08771-z>.

[7] R. K. Hammond, M. C. Pahl, C. Su, et al., “Biological constraints on GWAS SNPs at suggestive significance thresholds reveal additional BMI loci”, *Elife*, **2021**, *10*, e62206, <https://doi.org/10.7554/eLife.62206>.

[8] Z. Zhu, F. Zhang, H. Hu, et al., “Integration of summary data from GWAS and eQTL studies predicts complex trait gene targets”, *Nature Genetics*, **2016**, *48* (5), 481-487, <https://doi.org/10.1038/ng.3538>.
